# Different Disease Endotypes in Phenotypically Similar Vasculitides Affecting Small-to-Medium Sized Blood Vessels

**DOI:** 10.3389/fimmu.2021.638571

**Published:** 2021-02-22

**Authors:** Erin E. Gill, Maren L. Smith, Kristen M. Gibson, Kimberly A. Morishita, Amy H. Y. Lee, Reza Falsafi, Jinko Graham, Dirk Foell, Susanne M. Benseler, Colin J. Ross, Raashid A. Luqmani, David A. Cabral, Robert E. W. Hancock, Kelly L. Brown, The PedVas Initiative Investigators

**Affiliations:** ^1^Department of Microbiology and Immunology, University of British Columbia, Vancouver, BC, Canada; ^2^Department of Medical Genetics, University of British Columbia, Vancouver, BC, Canada; ^3^BC Children's Hospital Research Institute, Vancouver, BC, Canada; ^4^Department of Pediatrics, University of British Columbia, Vancouver, BC, Canada; ^5^BC Children's Hospital, Vancouver, BC, Canada; ^6^Department of Statistics and Actuarial Science, Simon Fraser University, Burnaby, BC, Canada; ^7^Department of Pediatric Rheumatology and Immunology, University Hospital Muenster, Muenster, Germany; ^8^Department of Pediatrics, Alberta Children's Hospital, Calgary, AB, Canada; ^9^Faculty of Pharmaceutical Sciences, University of British Columbia, Vancouver, BC, Canada; ^10^Nuffield Department of Orthopedics, Rheumatology and Musculoskeletal Sciences, University of Oxford, Oxford, United Kingdom; ^11^Centre for Microbial Diseases and Immunity Research, University of British Columbia, Vancouver, BC, Canada; ^12^Centre for Blood Research, University of British Columbia, Vancouver, BC, Canada

**Keywords:** vasculitis, neutrophils, transcriptome, inflammation, ANCA

## Abstract

**Objectives:** Chronic primary vasculitis describes a group of complex and rare diseases that are characterized by blood vessel inflammation. Classification of vasculitis subtypes is based predominantly on the size of the involved vessels and clinical phenotype. There is a recognized need to improve classification, especially for small-to-medium sized vessel vasculitides, that, ideally, is based on the underlying biology with a view to informing treatment.

**Methods:** We performed RNA-Seq on blood samples from children (n = 41) and from adults (n = 11) with small-to-medium sized vessel vasculitis, and used unsupervised hierarchical clustering of gene expression patterns in combination with clinical metadata to define disease subtypes.

**Results:** Differential gene expression at the time of diagnosis separated patients into two primary endotypes that differed in the expression of ~3,800 genes in children, and ~1,600 genes in adults. These endotypes were also present during disease flares, and both adult and pediatric endotypes could be discriminated based on the expression of just 20 differentially expressed genes. Endotypes were associated with distinct biological processes, namely neutrophil degranulation and T cell receptor signaling.

**Conclusions:** Phenotypically similar subsets of small-to-medium sized vessel vasculitis may have different mechanistic drivers involving innate vs. adaptive immune processes. Discovery of these differentiating immune features provides a mechanistic-based alternative for subclassification of vasculitis.

## Introduction

Vasculitis (1) is a group of complex rare diseases that are characterized by inflammation in the blood vessel walls. The disease can present in childhood and in adulthood, and can be life- and/or organ-threatening. The primary framework for classifying vasculitis syndromes is according to the predominant size of the involved vessels (small, medium, large), the clinical phenotype (pattern of organs affected), and histopathology of involved vessels (2–5). Recently, distinctive etiological/pathological processes have been incorporated in the classification framework; for example, an association with anti-neutrophil cytoplasmic antibodies (ANCA) against intracellular granule proteins proteinase-3 (PR3) and myeloperoxidase (MPO) enables classification of small-to-medium sized, ANCA-associated vasculitis (AAV) (3, 4, 6, 7).

AAV encompasses three specific diseases: microscopic polyangiitis (MPA), granulomatosis with polyangiitis (GPA) and eosinophilic granulomatosis with polyangiitis (EGPA). An absence of specific classification criteria for MPA and the considerable phenotypic overlap with GPA, however, makes it challenging to distinguish GPA and MPA (6–8). In adult clinical trials, they are frequently analyzed collectively for convenience (9), despite important clinical and biological differences that argue for tailored treatment. Specifically, some studies show that GPA has a more refractory and relapsing disease course than does MPA (10, 11), although in more recent studies the association with relapse seems to be stronger with the presence of PR3-ANCA. ANCA specificity (for PR3 or MPO) has been suggested as an alternative to the clinical phenotype classification, or as a “biomarker” in updated classification criteria for AAV. However, PR3-ANCA, despite being predominantly associated with GPA, is also present in one-quarter of patients with MPA. Similarly, MPO-ANCA is differentially, but not exclusively, associated with MPA (7, 12), and a proportion of patients with AAV do not have ANCA to either PR3 or MPO. It is also noteworthy that GPA and MPA have overlapping clinical features with polyarteritis nodosa (PAN) (a medium-sized vessel vasculitis), and other small-to-medium sized vessel vasculitides that remain “unclassifiable” according to existing classification criteria.

Here, we considered if classification of small-to-medium sized vessel vasculitides could be improved by a deeper understanding of the molecular events underlying the disease and distinct disease subsets. To explore this hypothesis, and despite the disease being especially rare in children compared to adults, we focused on a cohort of children and adolescents with small-to-medium sized vessel vasculitis for mechanistic discovery. The study of pediatric patients can be advantageous (13, 14); children have limited confounding disease comorbidities and may have more predominant genetic factors that lead to early disease manifestations, compared to adults that have multiple environmental factors contributing to the onset of disease, which, for vasculitis, typically occurs after 50 years of age. Using RNA-Seq on blood obtained from pediatric patients with different clinically defined subtypes of small-to-medium sized vasculitis, we were able to cluster patients into two groups with distinct transcriptomic profiles and associated immune processes. Individuals with adult-onset disease could also be categorized in a similar manner, together suggesting that both adult- and pediatric-onset small-to-medium sized vessel vasculitides within the same disease “category” due to overlapping clinical features, might have different endotypes (15, 16).

## Materials and Methods

### Participants

Patients described in this study were enrolled in the Pediatric Vasculitis Initiative (PedVas) (17, 18) and included children (18 yrs of age and younger) and adults with small-to-medium sized blood vessel vasculitis. Two pediatric cohorts were used for this study: Cohort 1 contained a total of 30 patients that contributed samples at diagnosis (*n* = 25) or relapse (*n* = 5); at the time of sample and data collection, disease activity was high (indicated by PVAS; see clinical data description) and this cohort was used for initial transcriptomic discovery. Pediatric Cohort 2 consisted exclusively of patients (n = 11) at relapse and was used to validate gene expression patterns observed in Cohort 1. Major relapse was defined as a new or recurrent appearance of life- or organ-threatening disease activity that occurs more than 18 months post diagnosis and requires a change in treatment. Adult participants with chronic primary vasculiltis were first enrolled in DCVAS, the Diagnostic and Classification Criteria for Vasculitis study.

### Clinical Data

Physicians collected data from pediatric participants (see Table 1 for Cohort 1 and Supplementary Table 1 for Cohort 2) (12, 17, 18) and entered it into A Registry of Childhood Vasculitis (ARChiVe), the RedCap data collection platform for PedVas. Generation of a pediatric vasculitis activity score (PVAS) (19) was a component of data entry; active and inactive disease was defined as a PVAS of > 2 and ≤ 2, respectively. The subtype of vasculitis was determined by a pediatric modified algorithm of the European Medicines Agency (EMA). ANCA status was reported by the participating site and validated in serum samples using a standard ELISA for anti-PR3 antibody (ORG518, Orgentec) and anti-MPO antibody (425-2380, BioRad). For adult patients, clinical data (Table 2) were collected through DCVAS. All DCVAS clinical data was reviewed independently by a panel of experts 6 months after the baseline assessment to provide a definitive, agreed diagnosis in accordance with the DCVAS protocol. For patients with GPA, MPA, and EGPA, there was ~25% rejection of the submitting physicians original diagnosis following the review.

**Table 1:** Characteristics and classification of Cohort 1 pediatric vasculitis patients.

**ID**	**^**a**^Endotype**	**^**b**^EMA**	**^**c**^ANCA**	**Time**	**Sex**	**^**d**^Organ system involvement**	**^**e**^PVAS**
C1 Patient 3	Endotype A	GPA	PR3	Diagnosis	Female	Skin, ENT, lung, CNS, MSK, renal	33
C1 Patient 23	Endotype A	GPA	PR3	Relapse	Female	ENT, lung, renal	8
C1 Patient 24	Endotype A	GPA	PR3	Relapse	Female	Skin, eye, ENT, lung, MSK, renal	19
C1 Patient 6	Endotype A	GPA	PR3	Diagnosis	Female	Skin, eye, ENT, lung, MSK, renal	30
C1 Patient 25	Endotype A	GPA	PR3	Diagnosis	Female	Lung, renal	19
C1 Patient 7	Endotype A	GPA	PR3	Diagnosis	Male	Skin, lung, MSK, renal	21
C1 Patient 17	Endotype A	GPA	PR3	Diagnosis	Male	ENT, lung, renal	26
C1 Patient 11	Endotype A	GPA	MPO	Diagnosis	Female	Skin, ENT, lung, renal	30
C1 Patient 22	Endotype A	GPA	MPO	Diagnosis	Female	Skin, lung, renal	20
C1 Patient 14	Endotype A	uAAV	PR3	Diagnosis	Male	Eye, ENT, renal	21
C1 Patient 21	Endotype A	uAAV	PR3	Diagnosis	Female	Lung, renal	23
C1 Patient 13	Endotype A	uAAV	MPO	Diagnosis	Female	Renal	18
C1 Patient 19	Endotype A	uAAV	MPO	Diagnosis	Male	Skin, ENT, lung	10
C1 Patient 10	Endotype B	GPA	MPO	Diagnosis	Female	Lung, MSK	10
C1 Patient 15	Endotype B	GPA	MPO	Diagnosis	Female	ENT, renal	17
C1 Patient 1	Endotype B	GPA	PR3	Diagnosis	Female	Skin, ENT, lung, renal	23
C1 Patient 8	Endotype B	GPA	PR3	Diagnosis	Female	Skin, lung, MSK, renal	21
C1 Patient 29	Endotype B	GPA	PR3	Relapse	Male	ENT, lung, renal	10
C1 Patient 20	Endotype B	MPA	MPO	Diagnosis	Female	Renal	12
C1 Patient 28	Endotype B	MPA	MPO	Diagnosis	Female	Skin, MSK, renal	16
C1 Patient 16	Endotype B	MPA	MPO	Diagnosis	Male	Skin, eye, renal	16
C1 Patient 12	Endotype B	MPA	PR3	Diagnosis	Male	Eye, renal	15
C1 Patient 4	Endotype B	PAN	NEG	Diagnosis	Female	Skin	9
C1 Patient 26	Endotype B	PAN	NEG	Diagnosis	Male	Skin, eye, ENT	12
C1 Patient 30	Endotype B	uAAV	MPO	Relapse	Female	Eye, renal	19
C1 Patient 9	Endotype B	UCV	NEG	Diagnosis	Male	Eye, MSK	5
C1 Patient 18	Endotype B	uAAV	PR3	Diagnosis	Female	ENT	7
C1 Patient 27	other	GPA	PR3	Diagnosis	Female	Skin, ENT, lung, MSK, renal	32
C1 Patient 2	other	GPA	MPO	Diagnosis	Male	Skin, lung, cardiac, renal	20
C1 Patient 5	other	uAAV	MPO	Relapse	Female	Lung	11

a *Hierarchical cluster (Endotype A, light grey; Endotype B, dark grey) based on RNA-sequence analysis*.

b *European Medicines Agency classification (GPA, blue; MPA, green; unclassified, white)*.

c *PR3 and MPO indicate positivity for, respectively, anti-PR3 (yellow) and anti-MPO (red) antibodies. NEG means that neither anti-PR3 nor anti-MPO antibodies were detected*.

d *ENT = ear, nose, throat; MSK = musculoskeletal; CNS = central nervous system*.

e *PVAS is the pediatric vasculitis activity score of disease activity at the time of sample collection*.

**Table 2:** Characteristics and classification of adult vasculitis patients.

**ID**	**^**a**^Cluster**	**^**b**^Physician diagnosis**	**^**c**^ANCA**	**Sex**	**^**d**^Organ systems involved**	**^**e**^BVAS**
Adult Patient 2	Adult Endotype A	GPA	PR3	Female	Systemic, skin, eyes, ENT, chest, abdominal, renal	45
Adult Patient 5	Adult Endotype A	GPA	PR3	Male	ENT, chest, renal	18
Adult Patient 3	Adult Endotype A	COMP/GBM	PR3 and GBM	Male	Systemic, ENT, renal	19
Adult Patient 4	Adult Endotype A	MPA	MPO	Female	Systemic, skin, abdominal, renal	26
Adult Patient 6	Adult Endotype A	MPA	MPO	Male	Systemic, skin, renal	17
Adult Patient 8	Adult Endotype B	MPA	PR3	Male	Lungs, kidneys	16
Adult Patient 9	Adult Endotype B	MPA	MPO	Female	Systemic, skin, eyes, abdominal, chest, renal	31
Adult Patient 7	Adult Endotype B	GPA	NEG	Male	Lungs	5
Adult Patient 10	Adult Endotype B	OSV	NEG	Male	Systemic, skin	9
Adult Patient 11	Adult Endotype B	EGPA	NEG	Male	ENT, chest, abdominal, neurological	25
Adult Patient 1	other	MPA	MPO	Male	Systemic, renal, ENT	18

a *Hierarchical cluster based on RNA-sequence analysis (Endotype A, light grey; Endotype B, dark grey)*.

b *Classification by expert consensus: (GPA, blue; MPA, green; EGPA, COMP and OSV are white) COMP/GBM = complex, anti-glomerular basement membrane disease, OSV = other small vessel vasculitis (leucocytoclastic cutaneous vasculitis)*.

c *PR3 and MPO indicate positivity for, respectively, anti-PR3 (yellow) and anti-MPO (red) antibodies. NEG means that neither anti-PR3 nor anti-MPO antibodies were detected*.

d *ENT = ear, nose and throat*.

e *BVAS is the Birmingham vasculitis activity score of disease activity*.

### RNA Sequencing and Analysis

Blood (2.5 ml) was collected from study participants in Tempus Blood RNA tubes (Applied Biosystems^TM^, CA, USA) at the time of diagnosis or flare (flare; ≥18 months post diagnosis with a major change in the PVAS and sustained escalation of treatment) when disease activity was high (pediatric vasculitis activity score [PVAS] range 5–33). Extracted RNA (Tempus Spin RNA Isolation Kit, Thermo Fisher) underwent PolyA enrichment (NEBNEXT Poly(A) mRNA magnetic isolation kit, New England BioSciences), RNA-Seq library preparation (75bp or 100bp single end, KAPA Stranded total RNA kit, Roche) and was sequenced on an Illumina Genome Analyzer IIx or an Illumina HiSeq 2500. Fastq files were checked for quality (FastQC v0.11.8 and MultiQC v0.8) and aligned to the human genome (Ensembl GRCh38.93) using STAR v2.6 (20). HTSeq-count (HTSeq 0.61p1) was used to generate read count tables (21). Read counts from globin genes were removed bioinformatically and batch correction for sequencing date [shown to contribute to variance by the R package eigenR2 (22)] was performed using the ComBat function of the SVA package (23). Raw RNA-Seq counts were normalized for library size and heteroskedasticity by variance stabilizing transformation (vst). Differential gene expression analysis was performed using DESeq2 package v1.14.1 (24, 25). Pathway over-representation analysis was conducted in the ReactomePA v1.26.0 package for R (26) and network visualization was conducted using NetworkAnalyst (27). Differentially expressed genes identified by Grayson et al. (28) in nasal brushings from adults with GPA (compared to controls) were obtained and analyzed with ReactomePA. Entrez IDs and Ensembl IDs were then used to compare microarray probeset labels (28) to RNA-Seq data.

### Statistics

Differential gene expression from DESeq2 analysis of RNA-Seq data was defined as a ≥ ± 1.5-fold change (FC) and ≤ 0.05 false discovery rate. Pathway enrichment analysis in ReactomePA used hypergeometric overlap and *p*-values were adjusted for multiple testing using false discovery rate. Significant enrichment was defined as a false discovery rate ≤ 0.05. Rotation gene set testing (ROAST) (29) and competitive gene set testing (CAMERA) (30) were used to determine whether the 20 gene signature, identified in pediatric patients at diagnosis, was also significantly different in pediatric patients in relapse and in adult patients. Clinical metadata (see Supplementary Table 2) were analyzed using the Description of Categories and Multiple Factor Analysis (MFA) functions from the FactoMineR package for R (31).

### Data Availability

RNA-Seq data have been submitted to the Gene Expression Omnibus data sharing repository, and are accessible through GEO Series accession number: GSE129752.

## Results

### Whole Blood Gene Expression Patterns Delineated Distinct Endotypes of Pediatric Small-to-Medium Sized Vessel Vasculitis

**Figure 1:**
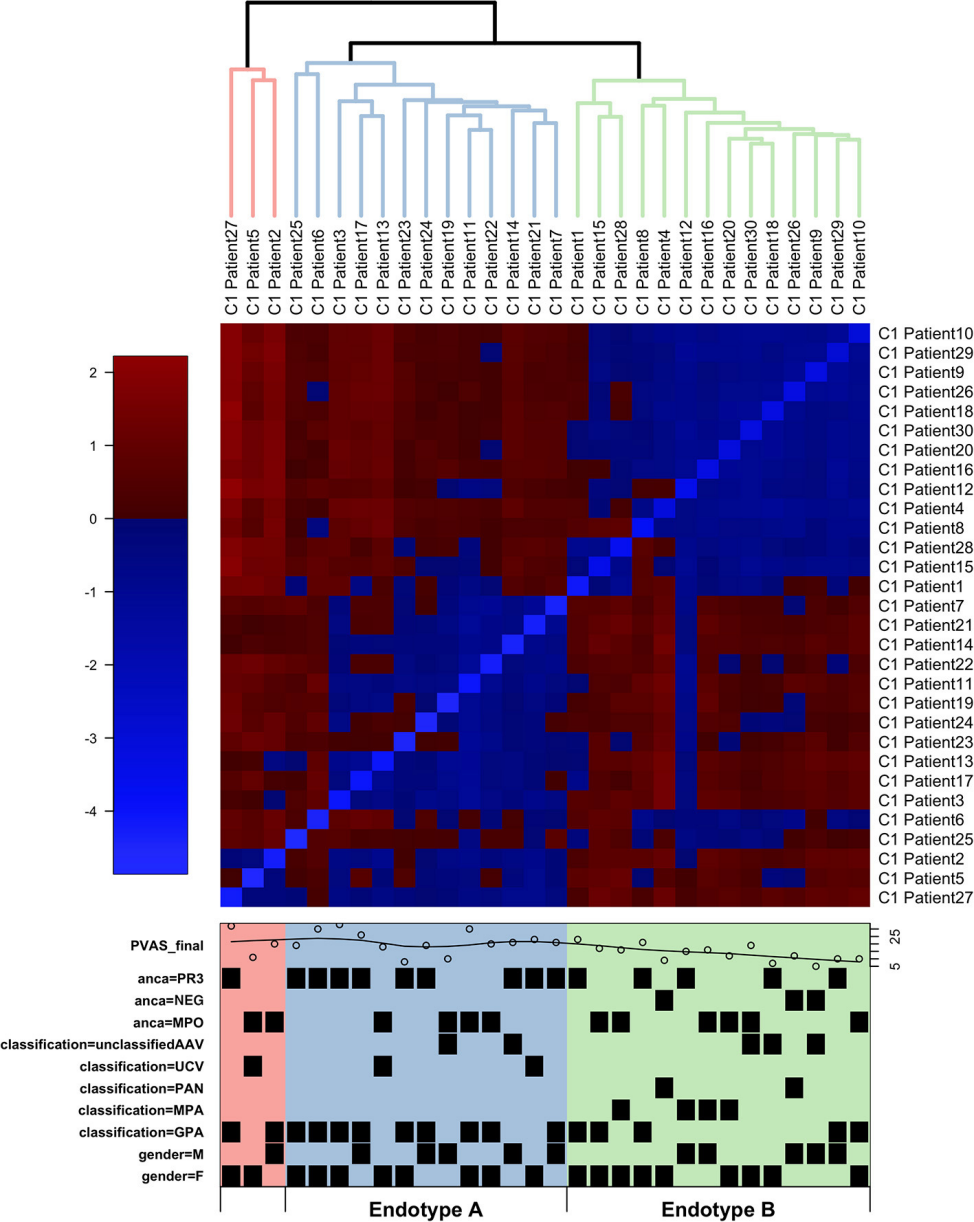
Hierarchical clustering of RNA-Seq data from children with small-to-medium sized vessel vasculitis. Hierarchical clustering (blue lines, Endotype A; green lines, Endotype B; red lines, “other”) and heatmap of normalized gene expression (variance stabilized counts) based on RNA sequencing of whole blood. Individual patient characteristics depicted in the lower box (also see Table 1): PVAS is the pediatric vasculitis activity score of disease activity at the time of sample collection; PR3 and MPO indicate positivity (or absence, NEG) for, respectively, anti-PR3 and anti-MPO antibodies; EMA classifications were uAAV, UCV, PAN, MPA, or GPA; M = male and F = female.

To identify the underlying molecules and pathways associated with different pediatric small-to-medium vessel vasculitides, we sequenced the whole blood transcriptomes of the 30 children and adolescents in Cohort 1. Study samples were collected from the majority at first disease onset and included EMA-defined subtypes of GPA (*n* = 16), MPA (*n* = 4), PAN (*n* = 2), unclassified ANCA-associated vasculitis (unclassified AAV; *n* = 7), and unclassified (ANCA-negative) vasculitis (UCV; *n* = 1). Unsupervised hierarchical clustering of global gene expression (without consideration of EMA classification) placed the samples into two major and one minor cluster (Figure 1 and Table 1).

Distinct patterns of whole blood gene expression and a total of 3,809 genes were differentially expressed (± 1.5 FC, FDR ≤ 0.05) between the two major clusters. One major cluster (A) contained samples (*n* = 13) from 4 male and 9 female patients: 9 with GPA, and 4 with unclassified AAV (uAAV). PR3-ANCA were present in 9 of the 13 ANCA-positive individuals in this group. The other major cluster (B) contained samples (*n* = 14) from 5 male and 9 female patients: 5 with GPA, 4 with MPA, 2 with PAN, 2 with uAAV, and 1 with UCV. All patients with MPA and PAN were in this cluster and MPO-ANCA were present in 7 of the 13 ANCA-positive individuals in this group.

Patients in cluster A had greater overall disease activity (Supplementary Table 2: mean PVAS = 21, *p*-value = 0.013 compared to cluster B mean PVAS = 14, *p*-value = 0.006), and specifically, higher disease activity in the respiratory domain (mean chest PVAS = 4, *p*-value = 0.022). These children (cluster A) were also diagnosed at significantly older ages (mean age = 14, *p*-value = 0.015) and showed significantly greater neutrophil counts than patients in cluster B (mean neutrophil count in Endotype A = 10.4, *p*-value = 0.018) (Supplementary Table 2 and Supplementary Figure 1).

These clusters (A and B) were consistent with the predominant EMA subtype in each cluster, that is, GPA in A and MPA/PAN in B, although overlap was observed especially for patients with GPA (Figure 1 and Table 1). These data together with the distinct patterns of whole blood gene expression associated with each major cluster suggests that the four EMA-defined (phenotypically classified) subtypes of small-to-medium sized vessel vasculitis in our cohort may fall under two major endotypes, A and B. The final, minor cluster contained three samples; 2 from patients with GPA and one from a patient with uAAV, and they could conceivably represent a rarer endotype.

### Neutrophil Degranulation and T Lymphocyte Activation Were Associated With Pediatric Vasculitis Endotype A and B, Respectively

**Figure 2:**
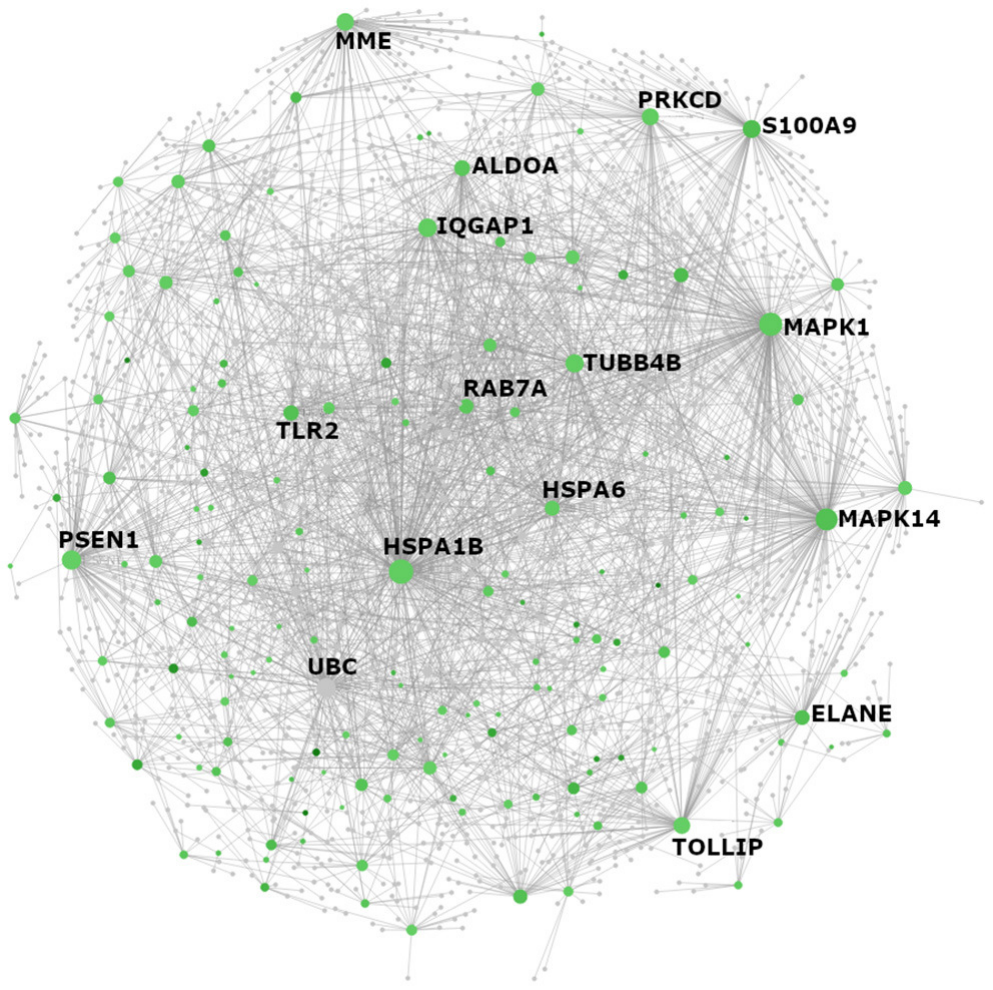
NetworkAnalyst visualization of Reactome pathway “neutrophil degranulation” genes with elevated expression in individuals from Endotype A. All genes with significantly higher expression in Endotype A that appeared in the Reactome PA category “neutrophil degranulation” (R-HSA-6798695) were visualized as a protein:protein interaction network using NetworkAnalyst. The color of each node reflects fold change between Endotypes A and B where green indicates higher expression in Endotype A. Gray nodes are genes that are not themselves differentially expressed (DE) but interact with other DE neutrophil degranulation genes. The size of each node (gene) reflects the number of documented interactions with other DE genes in the network. Thus, the largest circles have the greatest number of DE interacting partners and are predicted to be the most important regulators (“hub proteins”) of this network: inflammatory biomarker S100A8/A9, mitogen-activated protein kinases MAPK1 (ERK2) and MAPK14 (p38 MAPK), heat shock protein HSPA1B, the lysosome-associated glycoprotein LAMP2, tubulin TUBB4B, and Toll-like receptor protein TLR2 and its regulator/adaptor TOLLIP.

Among the 3,809 differentially expressed (DE) genes between Endotype (cluster) A and Endotype (cluster) B, a total of 2,217 genes were expressed higher in Endotype A (relative to Endotype B) and 1,592 genes were expressed higher in Endotype B (relative to Endotype A). Genes with higher expression in Endotype A were involved in Toll-like receptor (TLR) signaling, interleukin signaling, Rho GTPase signaling, cellular senescence, and neutrophil degranulation (Supplementary Figure 2 and Supplementary Table 3A). Within the latter pathway, 178 of the 479 known neutrophil degranulation genes (~37%) were differentially expressed (FDR adjusted *p*-value for significance of association = 1.1 × 10^−56^). Functional interactions between the encoded proteins are shown in a protein:protein interaction network in Figure 2. Related, gene expression of biomolecules (including 16 histone genes, S100A8, S100A9, and PADI4) that are associated with neutrophil extracellular traps (NETs) (32) were higher (FDR <0.05) in Endotype A compared to Endotype B.

**Figure 3:**
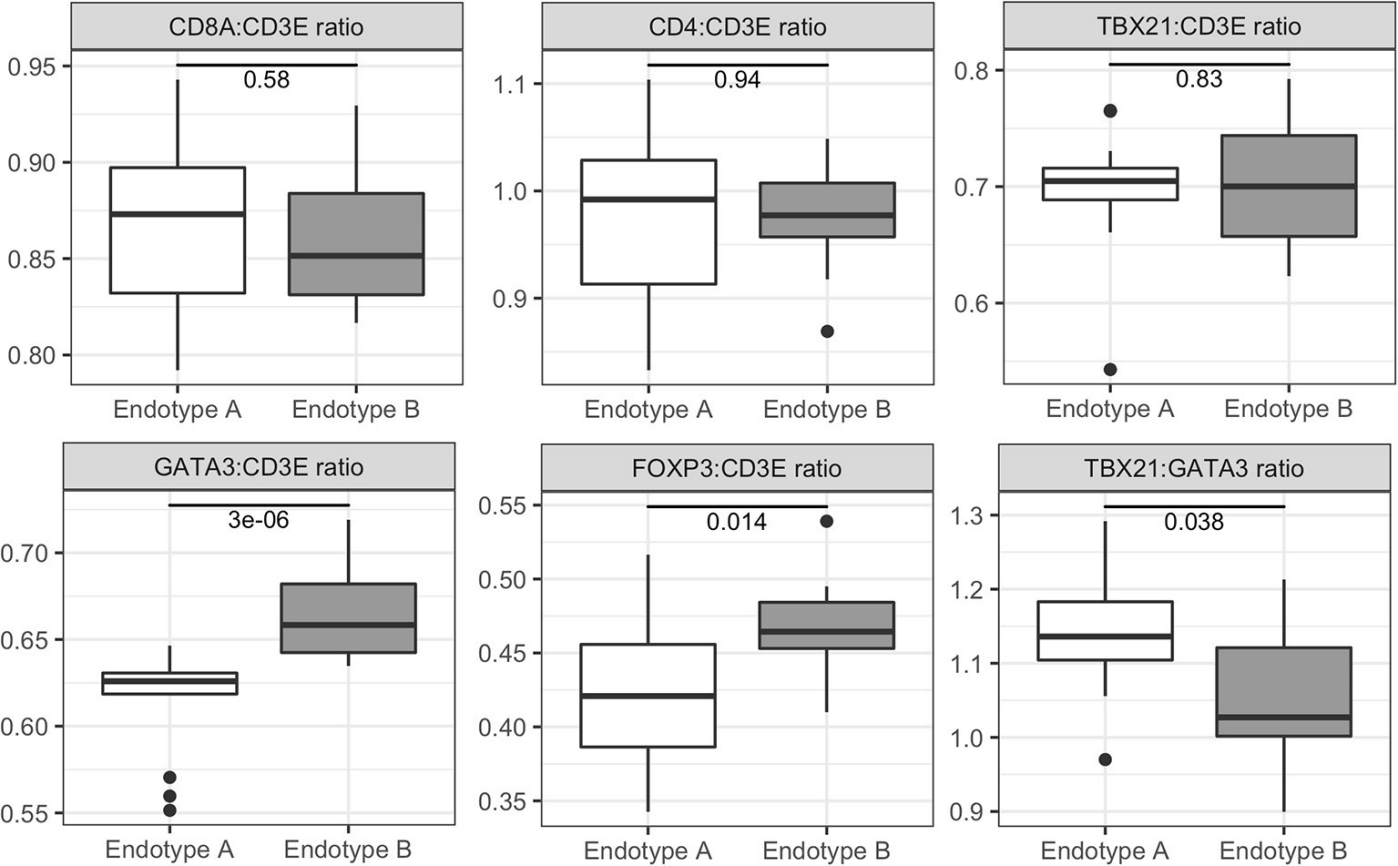
Expression ratios of T cell related genes for pediatric vasculitis Endotypes A and B. Relative abundance and ratios (y-axis) of the expression of genes for T cell markers in Endotype A and Endotype B patients (x-axis). T cell marker genes included CD3E (present in all T cells), CD8A (CD8+ cells), CD4 (CD4+ cells), TBX21 (Th1 cells), GATA3 (Th2 cells), FOXP3 (Tregs), and RORC (Th17 cells). Gene expression ratios were calculated from variance stabilized counts. Significance of the ratio between the clusters is reported within each boxplot and was determined by the Wilcoxon Rank Sum test. Additional data shown in Supplementary Figure 3.

In contrast, genes with elevated expression in Endotype B were associated with T cell receptor (TCR) signaling, RNA processing, interferon (IFN) signaling and the differentiation and regulation of T cells and NK cells (Supplementary Figure 2 and Supplementary Table 3B). Dysregulated pathways in this cluster included: phosphorylation of CD3 and TCR zeta chains, PD-1 signaling, co-stimulation by the CD28 family, TCR signaling, and immunoregulatory interactions between lymphoid and non-lymphoid cells. Children in Endotype B had a moderate but significantly lower ratio of Th1:Th2 cells marker expression (TBX21:GATA3) (*p*-value = 0.038 by Wilcoxon test) compared to Endotype A (Figure 3 and Supplementary Figure 3), and a much overall higher ratio of expression of the Th2 marker GATA3 (*p*-value = 3.0 × 10^−6^). Individuals in Endotype B also had a higher expression ratio of the Treg marker FOXP3 (*p*-value = 0.014).

### Endotypes Present at Diagnosis Also Defined Inflammatory Mechanisms at Relapse

To determine if the endotypes (and immunologic pathways) identified in Cohort 1 were associated with subsequent relapses in disease post diagnosis, we performed RNASeq on samples collected from a smaller cohort of pediatric patients (Cohort 2, *n* = 11) during a major disease relapse. Using unsupervised hierarchical clustering of RNASeq data as was done with Cohort 1, patients in Cohort 2 also sorted into two major clusters, ‘Relapse cluster 1' and ‘Relapse cluster 2'. Two outlier samples did not fall into either cluster and did not cluster with each other (Supplementary Figure 4A).

Relapse cluster 1 contained 4 patients: 2 with GPA, 1 with uAAV, and 1 with UCV. ANCA status was unknown for 1 patient and the remaining 3 patients were all positive for PR3-ANCA. Relapse cluster 2 contained 5 patients: 3 with GPA and 2 with MPA. This cluster contained 4 ANCA-positive patients: 3 with MPO-ANCA and 1 patient with both MPO- and PR3-ANCA. We noted that all patients (4/4) in relapse cluster 1 were female, and suggest that this is a reflection of Cohort 2 being small, and predominantly female (7/11 patients); a separation of sexes between Endotypes A and B was not observed in Cohort 1, which was larger and balanced between male and female patients.

Using the more than 1,000 genes that were differentially expressed between relapse clusters 1 and 2, we performed Reactome analysis and compared pathway enrichment in the “relapse clusters”, (Supplementary Figure 4B and Supplementary Table 4) to those in the “diagnosis clusters” (i.e., Endotype A and Endotype B from Cohort 1). Our results revealed an overlap in enriched pathways between relapse cluster 1 and Endotype A, and between relapse cluster 2 and Endotype B, suggesting that the same driving mechanisms (behind the distinct Endotypes) are present at diagnosis and disease relapse.

### Pediatric Derived Molecular Pathways and Endotypes Defined Adult-Onset Small-to-Medium Sized Vessel Vasculitis

Unsupervised hierarchical clustering of RNASeq data from a small subset of adult vasculitis patients (*n* = 11, Table 2) also sorted into two distinct endotypes (Adult Endotype A and Adult Endotype B; Supplementary Figure 5A). Adult Endotype A contained 5 patients: 2 with MPA, 2 with GPA, and 1 with complex vasculitis (labeled COMP/GBM). Of these, 2 were positive for MPO-ANCA and 3 for PR3-ANCA. Adult Endotype B also included 5 patients: 1 with GPA, 1 with eosinophilic GPA (EGPA), 2 with MPA, and 1 with leucocytoclastic cutaneous vasculitis (labeled OSV). Three adult Endotype B patients were ANCA negative, 1 was positive for MPO-ANCA, and 1 was positive for PR3-ANCA. A total of 1,682 genes were differentially expressed between Adult Endotypes A and B. Similar to the pediatric endotypes, Adult Endotype A was enriched for neutrophil degranulation pathways, IL-4 and IL-13 signaling, and antimicrobial peptides, while Adult Endotype B was enriched for T cell receptor signaling, IL-2 signaling, CD28 dependent PI3K/AKT signaling, and translocation of ZAP-70 to the immunological synapse (Supplementary Figure 5B).

Due to the small cohort size, we also compared our results to a transcriptomic study by Grayson et al. (28) that identified 339 genes significantly DE in nasal brushings from adult GPA patients compared to healthy controls. Of these DE genes, 141 were highly expressed in pediatric Endotype A (Fisher's Exact test: *p*-value = 2.2 × 10^−16^, odds ratio = 7.6), and 62 were also highly expressed in Adult Endotype A (Fisher's Exact test: *p*-value = 2.2 × 10^−16^, odds ratio = 5.2). An overlap in Reactome pathways enriched in the nasal brushing dataset and Endotypes identified from our dataset were also observed (Supplementary Figure 5C).

Together, these findings indicate potentially common mechanisms between pediatric and adult vasculitis endotypes and suggest that gene signatures and disease processes in affected tissues may be evident in blood.

### Differential 20-Gene Signature Defines Pediatric and Adult Vasculitis Subtypes

**Figure 4:**
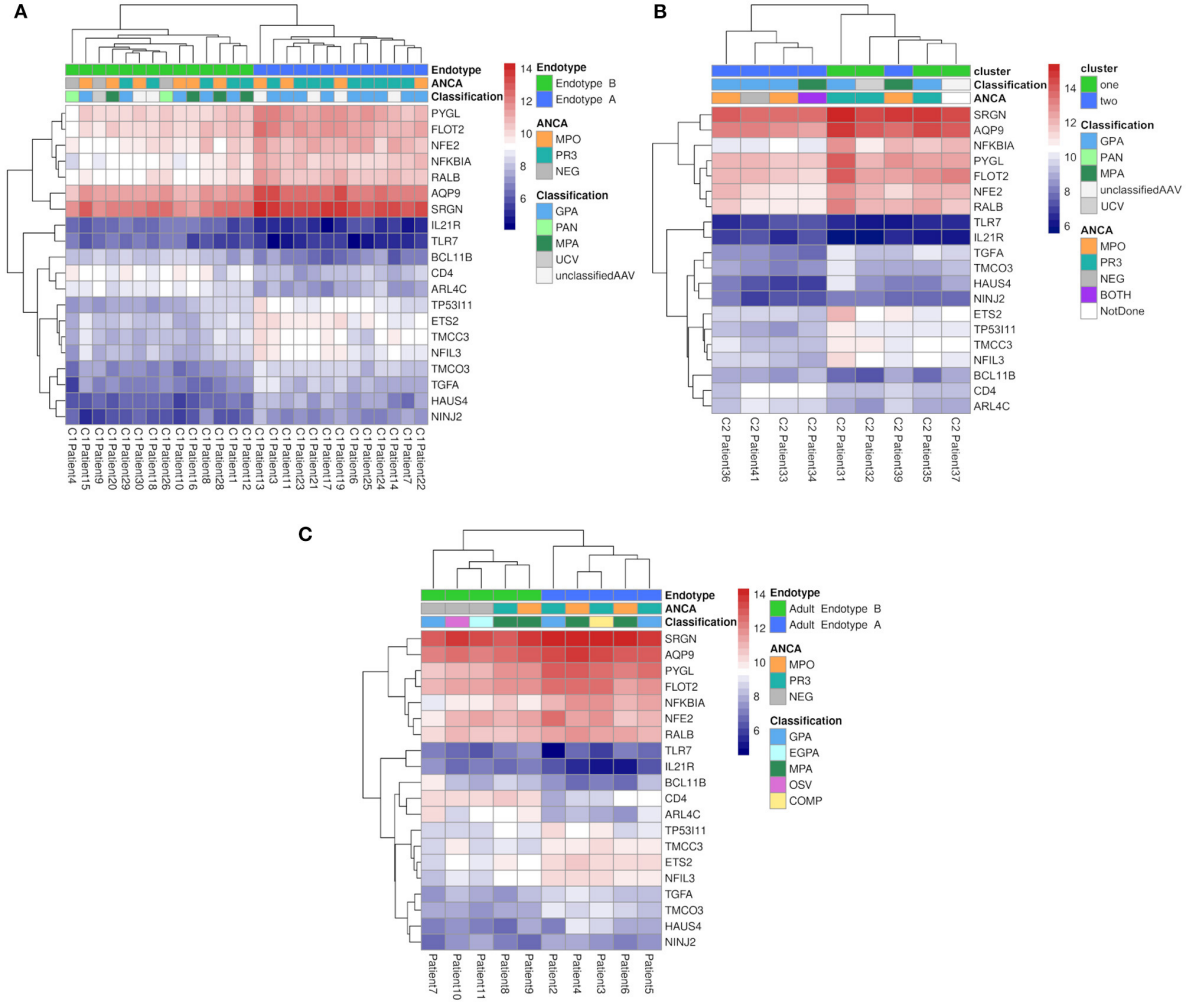
Hierarchical clustering of vasculitis patients based on expression data from 20 genes. Hierarchical clustering (hclust function of R) was performed using the average linkage method with Euclidean distances calculated from vst counts. The variance for all genes (as batch corrected vst counts) was calculated separately for major clusters. Genes with the lowest within-group variance, an absolute FC ≥ 2 between the clusters, the 50% least variable genes in both clusters, and an overall average count of at least 100 were selected and assessed for their ability to separate samples into the same clusters as the entire gene dataset. The heatmap represents expression (variance stabilized counts) of each of 20 genes (y-axis, right) that were (a) maximally divergent between Endotypes A and B (i.e.: significantly DE with a FDR adjusted *p*-value <0.05 and fold change >1.5) and (b) had low variance within each cluster to minimize the margin of error for testing individual samples. **(A)** Pediatric Cohort 1 patients (*n* = 27, x-axis) in Endotypes A (blue) and B (green). **(B)** Pediatric Cohort 2 patients (*n* = 9, x-axis) in relapse Endotypes A (blue) and B (green). **(C)** Adult patients (*n* = 10, x-axis) in Adult Endotypes A (blue) and B (green).

The differential gene expression patterns in Endotypes A and B, consistent in both the pediatric and adult cohorts, suggested fundamentally different disease mechanisms associated with each endotype. We therefore asked whether a small set of DE genes could reliably separate individual samples into the respective Endotypes (A and B). A 20 gene signature was identified from pediatric Cohort 1 (see Figure 4 and Materials and Methods) that, using hierarchical clustering, placed all samples within the same pediatric Endotypes as the full RNA-Seq dataset (Figure 4A and Supplementary Figure 6).

This 20 gene signature, when applied to pediatric Cohort 2 (relapse samples; Figure 4B) and the adult cohort (Figure 4C), also separated samples into the same Endotypes A and B as the entire RNA-Seq dataset with the exception of only 2 samples from the relapse cohort. The application of gene set significance testing, using both ROAST and CAMERA, showed that this 20 gene signature was also significantly differentially expressed between Endotypes A and B at relapse (ROAST *p*-value = 6.6 × 10^−03^, CAMERA *p*-value = 1.1 × 10^−03^). Similarly, the gene sets were also highly significantly different between endotypes (ROAST *p*-value = 4.10 × 10^−03^, CAMERA *p*-value = 7.72 × 10^−04^) in the adult cohort. Among the 20 gene signature identified in both pediatric and adult RNA-Seq data, 25% (5/20) of these genes were differentially expressed (28) in nasal brushings from adults with GPA.

## Discussion

Despite step-wise improvements in classification criteria for small-to-medium sized vessel vasculitis, the ability to use current classification systems to accurately diagnose, prognosticate and tailor treatment remains limited due to overlapping clinical features, unclassifiable patients and variable disease trajectories/outcomes of patients classified as having the same disease. For pediatric patients, classification is even more challenging. Even relatively recent pediatric adaptations of the American College of Rheumatology (ACR) criteria (originally based on adult data) (3, 4) fail to uniquely classify 25% of pediatric patients (12).

In this study, we investigated a biological basis to differentiate small-to-medium sized vessel vasculitis in children. Our results demonstrate that the majority of patients fell into two primary groups with distinctive gene expression patterns and clinical phenotypes that were predominantly, but not exclusively, either GPA or MPA. The groups differed in the expression of approximately one-third of all expressed human genes and could be discriminated based on the differential expression of just 20 “biomarker” genes. These transcriptome-based signatures, which were elucidated from predominantly disease-onset (diagnosis) pediatric samples, were also found in a small cohort of pediatric patients experiencing a relapse in disease, and in a small cohort of adult patients at diagnosis of a variety of clinically-defined vasculitides affecting small-to-medium sized vessels.

The concept that mechanistic differences may enable separation of complex diseases into different “endotypes” has been demonstrated in other chronic inflammatory diseases, for example, asthma (15) and diabetes (16) that have a singular “disease category” yet intrinsic heterogeneity in symptoms and outcomes. In our cohort, the transcriptome-defined endotypes were not significantly associated with MPO/PR3 ANCA positivity or EMA classification for GPA, indicating differentiating biological factors between endotypes that are not apparent based on current clinical classification or ANCA status. Of interest, unbiased cluster analysis of clinical metadata, including data used in the EMA classification algorithm, organized patients in our cohort into two “new” clinically-defined groups (Supplementary Figure 7) that had substantial overlap with RNASeq data-defined endotypes. In one clinical cluster, 10/13 patients were associated with Endotype A (correlation analysis p-value = 0.0018), and in the other clinical cluster, 12/17 patients were associated with Endotype B (correlation analysis *p*-value = 0.0037). Although a much larger cohort is required for validation, the data suggest that these underlying biologic mechanisms (endotypes) might each associate with a unique clustering of patients according to clinical symptoms.

Endotype A was associated with pathways reflecting neutrophil degranulation while Endotype B demonstrated a gene expression pattern indicative of T cell activation. In AAV, both innate and adaptive immune processes are, as the name suggests, involved in disease pathogenesis (i.e., by the action of autoantibodies against neutrophil proteins). Our evidence however, suggests that these different arms of the immune system predominate in different subsets of patients, as opposed to operating in concert across all individuals regardless of subtype, and seemingly independent of ANCA specificity.

Neutrophil degranulation, a common feature of many inflammatory disorders, including severe asphyxia in asthma, acute lung injury, rheumatoid arthritis, and septic shock (32), was associated with Endotype A. The gene encoding a protein from the neutrophil degranulation pathway, glycogen phosphorylase L (PYGL), was one of the top 20 most significantly DE genes between the endotypes (*p*-value = 1.85 × 10^−07^, mean fold change of genes in pathway = 2.17). Consistent with the role of neutrophil degranulation in lung disease, Endotype A patients had higher component PVAS for respiratory involvement (chest score: Supplementary Table 2). In contrast, there was no significant association between either endotype and renal-specific PVAS despite a large majority of patients with GPA and MPA having renal involvement. Moreover, pulmonary disease, and specifically, the involvement of granulomatous inflammation, occurs more frequently (twice as often) in GPA compared to MPA. Neutrophil degranulation can lead to neutrophil extracellular traps (NETs) (32, 33) that play a role in the capture and killing of bacteria and have been described in adult AAV (34, 35). NETs contain a variety of biomolecules (36) including DNA, histone proteins, S100 proteins, MPO and PR3. Consistent with the role of NETs in bacterial infections, genes involved in the recognition and uptake of bacteria, including TLR- and NOD-like receptor signaling, and FcγR-mediated phagocytosis were enriched in Endotype A; notably FcγRIIA and the phagocytic pathway downstream of this receptor were significantly (*p*-value = 0.025) enriched in Endotype A.

Endotype B contained all MPA/PAN patients, which is noteworthy given that MPA and PAN, prior to the identification of ANCA, were considered manifestations of the same disease in different sized blood vessels. Patients within Endotype B differentially expressed many genes associated with T cell receptor (TCR) signaling and the differentiation and regulation of T cells and NK cells. T cell surface protein CD4 was one of the top 20 DE genes showing higher expression in Endotype B. The expression of genes in the MHC class II pathway responsible for CD4^+^ T helper cell activation were also elevated. The MHC class II pathway can be triggered either by endocytosed antigens or misfolded proteins (autoantigens) as is the case with certain types of arthritis (37, 38). These are interesting observations, since Th2 and Treg cells are involved in the prevention of autoimmune diseases (39), suggesting that the mechanism of pathogenesis in Endotype B might trigger immune regulatory responses to a greater extent. Given that specific Th2 populations can play a role in several inflammatory diseases [e.g., ulcerative colitis (40), chronic allergic inflammation (41) and eosinophilic gastrointestinal disease (42)], inflammation in patients associated with this Endotype could also be driven by a similar mechanism involving highly activated T cells, and an imbalance of Th2 cells that is pathogenic.

Our results also demonstrated that pediatric gene expression patterns involving neutrophil degranulation and T cell activation were present in blood samples from adults with vasculitis and showed significant overlap with findings from a published study of gene expression in nasal tissue from adults with GPA (28). The latter observation is consistent with the notion that inflammatory mechanisms in AAV are independent of the organ systems involved (43).

It is important to note that therapy was initiated in the majority of patients prior to sample collection and many treatments, notably corticosteroids, can influence gene expression. The nature of treatment, dose and route of administration varied considerably among patients and was consistent with our previous report of treatment variability in clinical practice (44). Despite this, it should be noted that no association was observed between endotype and receipt of corticosteroids by multi-factor analysis (31) (Supplementary Table 2). In contrast, we found a significant correlation between patients in Endotype B and the receipt of non-biologic immunosuppressive treatment. However, it would be difficult to conclude that these treatments are driving differences in gene expression between groups given that factors other than the nature of the disease, including cost, accessibility of drugs, and physician experience influence treatment choice (44). It is also equally likely that inflammatory mechanisms, as observed by differences in gene expression, respond to different pharmacological agents, leading to improved patient outcomes and preferred use by physicians.

In summary, we have classified pediatric and adult patients with small-to-medium sized vessel vasculitis (AAV, PAN and unclassifiable disease) into two distinct endotypes based on whole blood gene expression profiling. These data may argue for categorization of vasculitides based on biologic mechanism, however it remains to be proven if transcriptome-defined groups have clinical utility. Exploration of links between treatment outcomes particularly relapsing or refractory disease, and the pathogenic mechanisms identified by transcriptomics, will be the subject of future analyses of pediatric and adult vasculitis patients.

## Data Availability Statement

The datasets presented in this study can be found in online repositories. The names of the repository/repositories and accession number(s) can be found in the article.

## Ethics Statement

The study protocol was approved by the Children's and Women's Research Ethics Board of the University of British Columbia [H12-00894] and the respective ethical committees or IRBs at participating PedVas sites. Written informed consent and assent/consent, respectively, was obtained from parents and from pediatric patients. Adults with chronic primary vasculitis were first enrolled in DCVAS, the Diagnostic and Classification Criteria for Vasculitis study. The DCVAS study consent form explicitly included an invitation for patients to participate in other research studies, subject to the patients consenting to those other studies, as was the case with inclusion of any adult samples in the pediatric vasculitis study.

## Author Contributions

EG and MS: bioinformatics analysis and manuscript preparation. KG and AL: data analysis and final revision of manuscript. RF: RNA sequencing. CR and JG: experimental design and final revision of manuscript. DF, SB, and KM: clinical care of patients, clinical data collection and entry, and final revision of the manuscript. PedVas Initiative Investigators: clinical care of patients and clinical data collection and entry. RL: clinical care of patients, clinical data collection and interpretation, and final revision of the manuscript. DC: study design, clinical care of patients, clinical data collection and interpretation, and final revision of the manuscript. RH: study design and bioinformatics data oversight and interpretation and manuscript preparation. KB: study design, sample collection, and clinical and bioinformatic data interpretation and manuscript preparation. All authors read and approved the final manuscript.

## Conflict of Interest

The authors declare that the research was conducted in the absence of any commercial or financial relationships that could be construed as a potential conflict of interest.

## Abbreviations

AAV: ANCA-associated vasculitis
ANCA: anti-neutrophil cytoplasmic antibody
ARChiVe: A registry for childhood vasculitis
BVAS: Birmingham vasculitis activity score
DCVAS: diagnostic and classification criteria for vasculitis
DE: differentially expressed
EMA: European medicines agency
FDR: false discovery rate
GPA: granulomatosis with polyangiitis
MPA: microscopic polyangiitis
MPO: myeloperoxidase
PAN: polyarteritis nodosa
PR3: proteinase 3
PVAS: pediatric vasculitis activity score
UCV: unclassified vasculitis.
