# Supplementary material for: Different Disease Endotypes in Phenotypically Similar Vasculitides Affecting Small-to-Medium Sized Blood Vessels
Source: Front Immunol. 2021 Feb 22;12:638571. doi: 10.3389/fimmu.2021.638571 (PMC7937946; doi:10.3389/fimmu.2021.638571)
Supplement: Supplementary file 1 [file Data_Sheet_1.docx]

**Supplementary Materials**


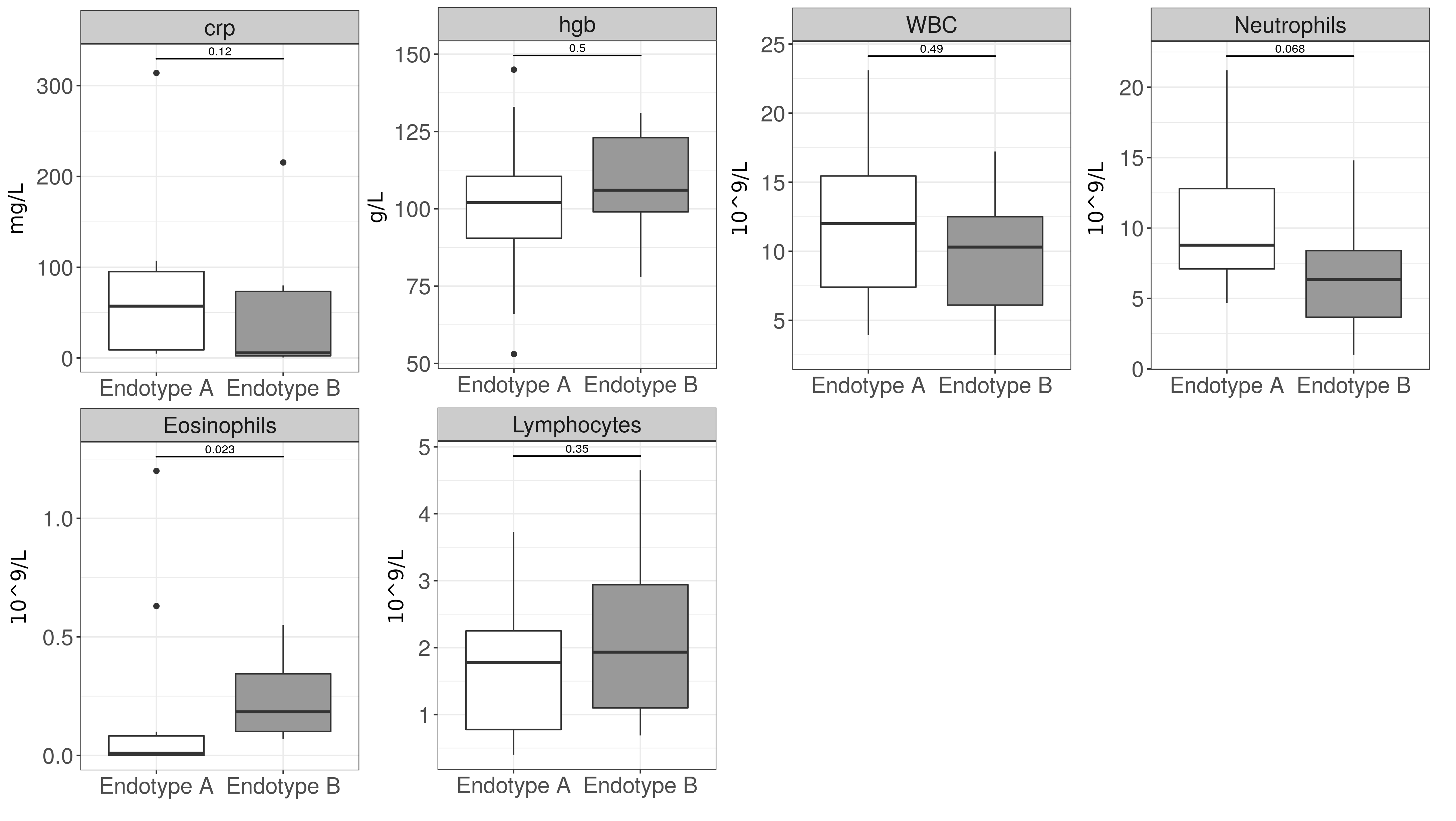

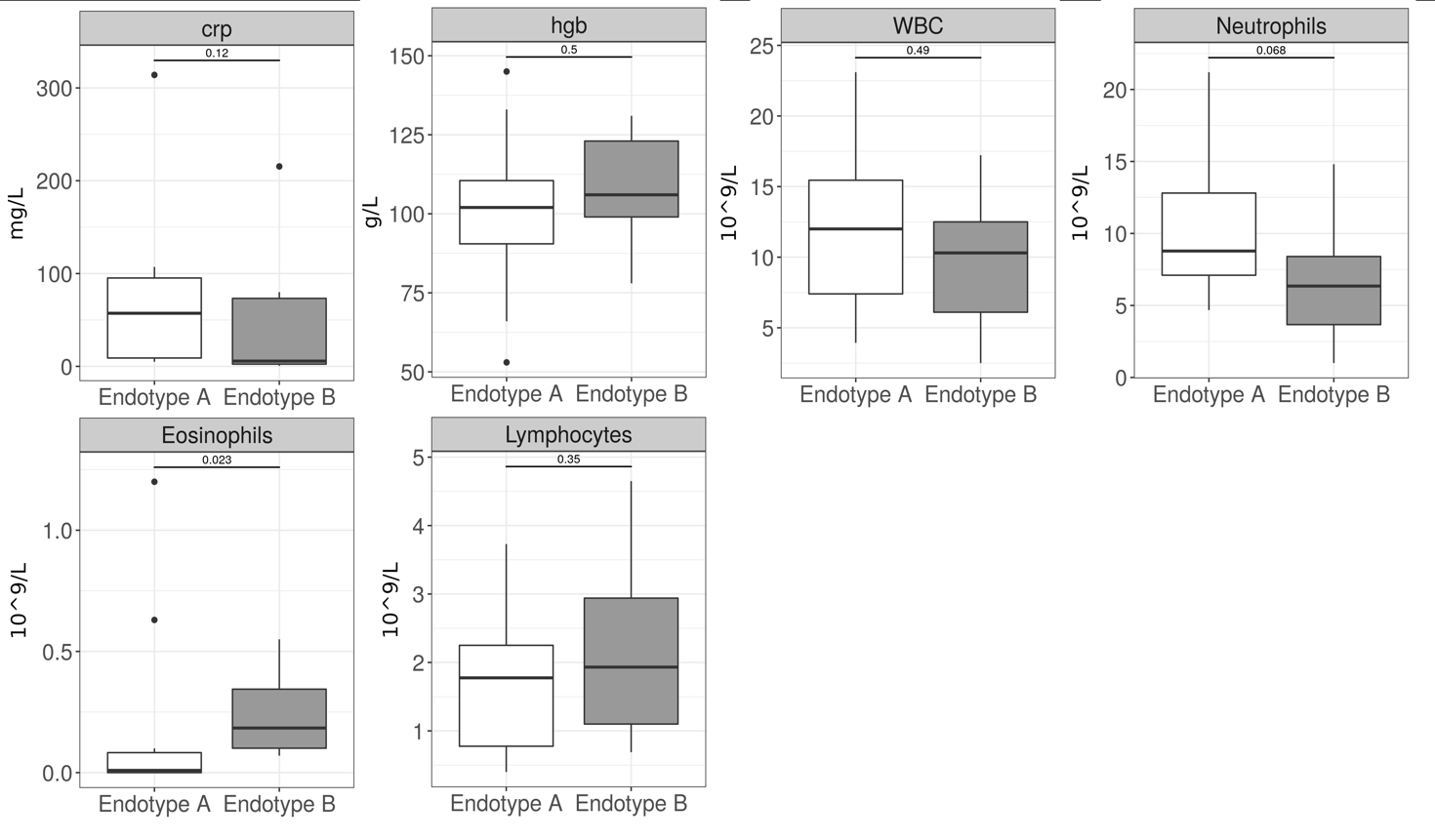


**Supplementary Figure 1: Standard clinical measures in pediatric patients, separated by endotype.** Boxplots show (y-axis) the mean standard deviation and range of concentrations of c-reactive protein (crp) and hemoglobin (hbg), and absolute blood counts of white blood cells (WBC), neutrophils, eosinophils and lymphocytes in samples (x-axis) belonging to Endotype A (n = 13) and Endotype B (n = 14)**.**


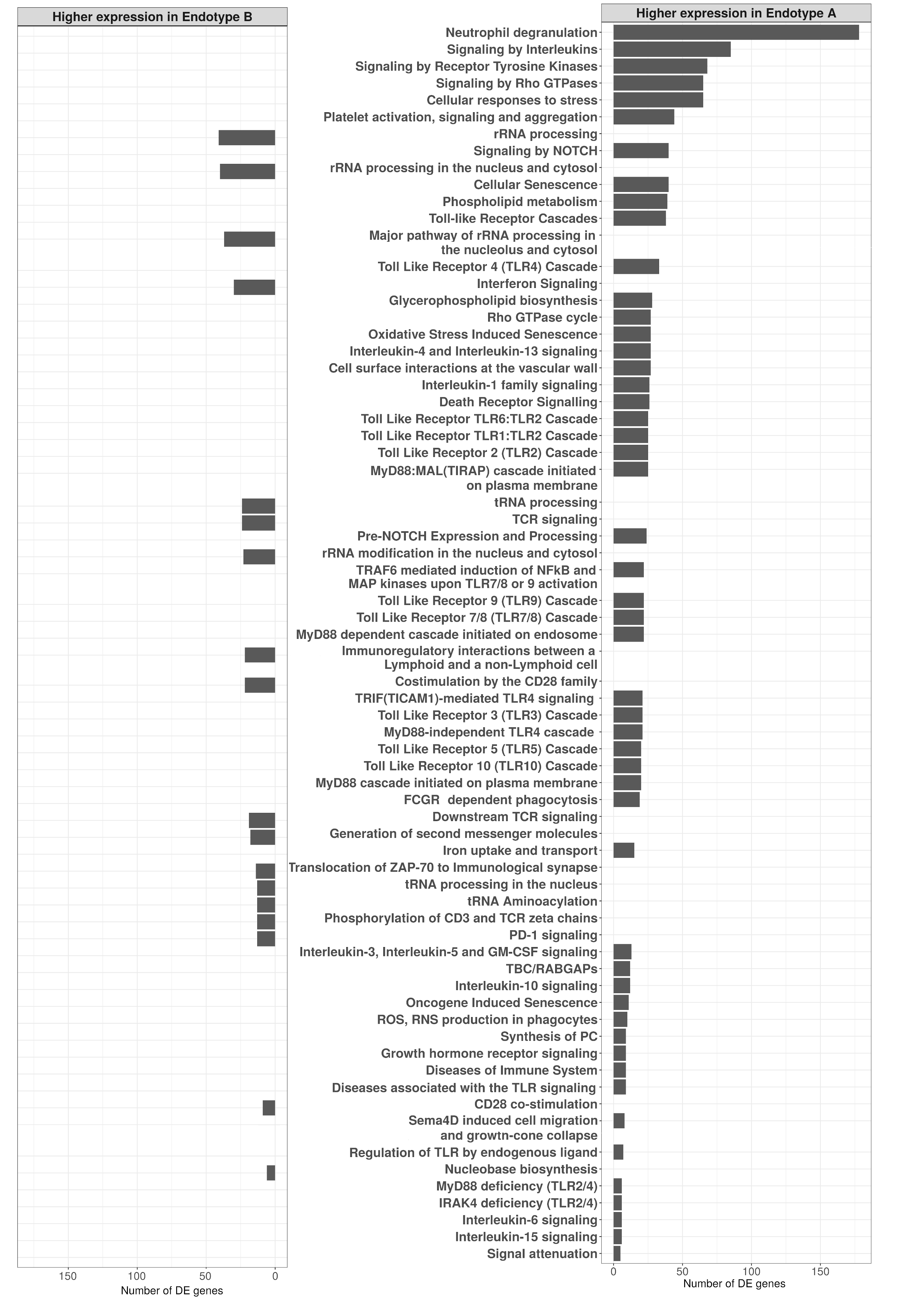


**Supplementary Figure 2: Dysregulated pathways in pediatric vasculitis patient Endotypes A and B.** Reactome pathways enriched for differentially expressed genes (x-axis, total number of DE genes, FDR < 0.05, and fold change > 1.5) associated with Endotype A (right) and Endotype B (left).

**
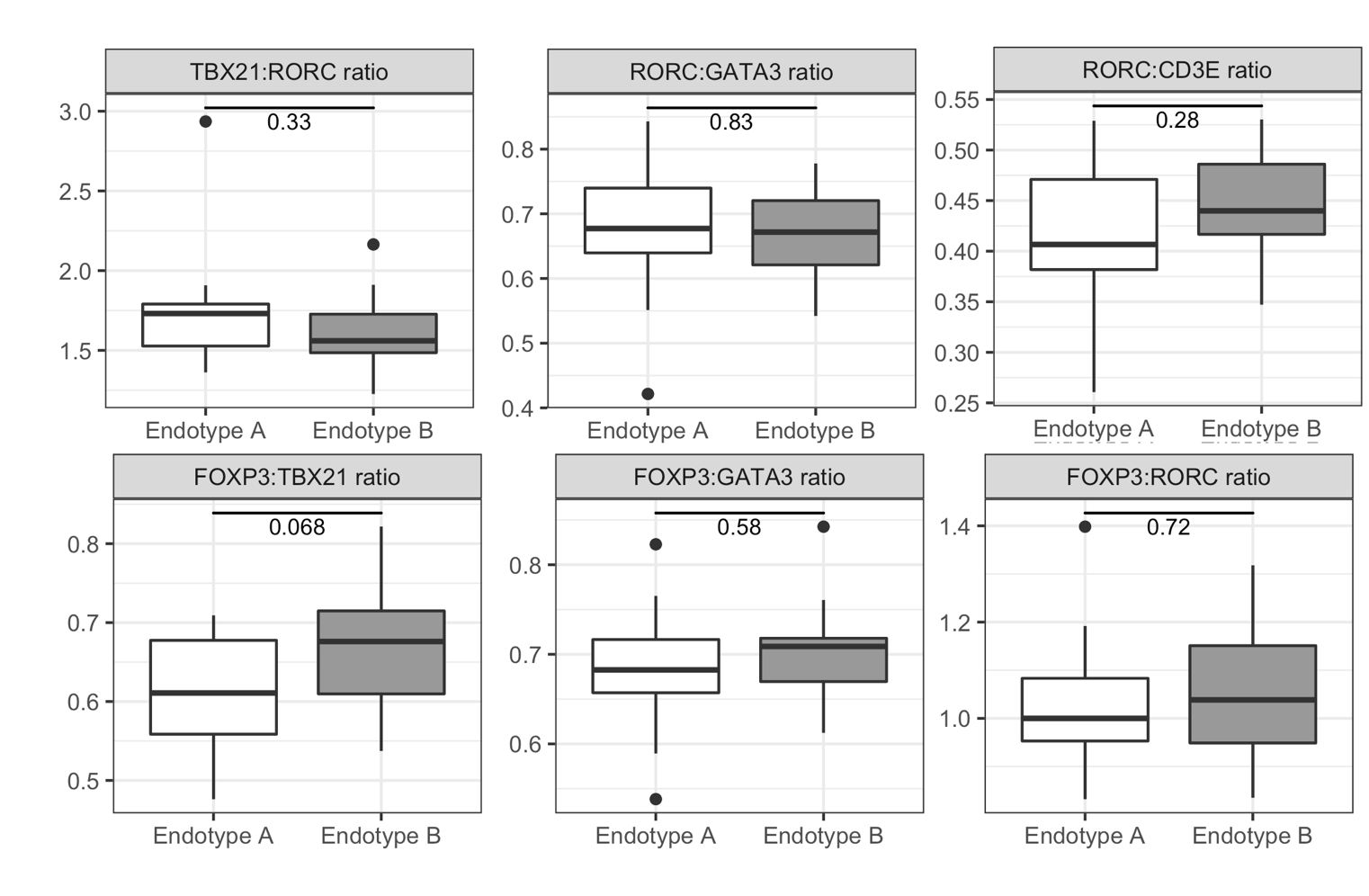
**

**Supplementary Figure 3: T cell subtype-associated gene expression ratios in pediatric Endotypes A and B.** Relative abundance and ratios (y-axis) of T cell markers at the mRNA level in Endotype A and Endotype B patients (x-axis) calculated from the normalized expression (variance stabilized counts) of genes CD3E (present in all T cells), CD8A (CD8+ cells), CD4 (CD4+ cells), TBX21 (Th1 cells), GATA3 (Th2 cells), FOXP3 (Tregs), and RORC (Th17 cells). Significance of the ratio between the clusters is reported within each boxplot and was determined by Wilcoxon Rank Sum test. Additional data shown in Figure 3.

**
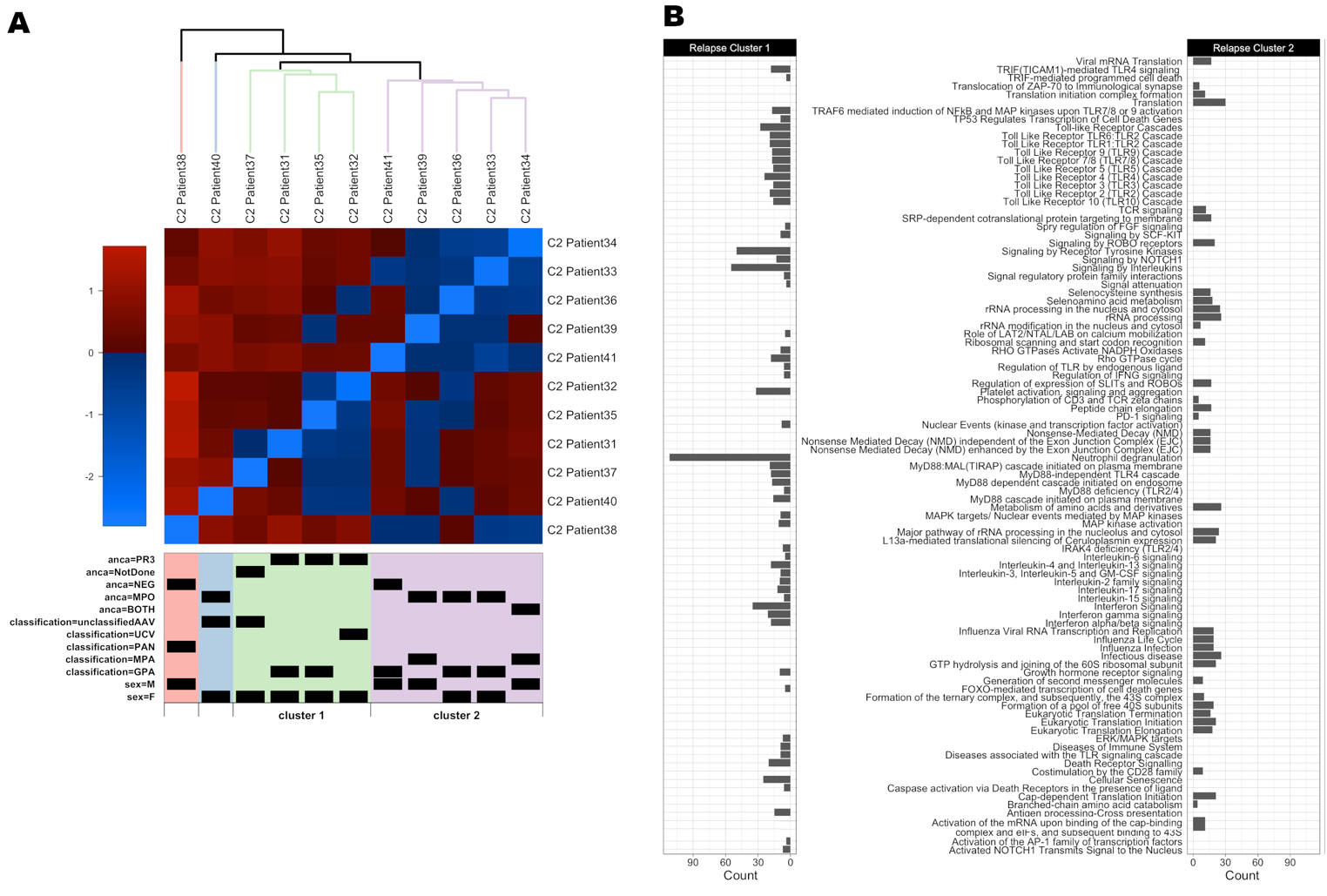
**

**Supplementary Figure 4:**  **Hierarchical clustering and dysregulated pathways in children with small-to-medium sized vessel vasculitis in relapse (Cohort 2). (A)** Hierarchical clustering and heatmap of normalized gene expression (variance stabilized counts) based on RNA sequencing of whole blood in 11 children and adolescents (Supplementary Table 1) with vasculitis at the time of a major disease flare. (**B)** Reactome pathways enriched for differentially expressed genes (x-axis, total number of DE genes, FDR < 0.05, and fold change > 1.5) associated with relapse cluster 1 (green in A) or relapse cluster 2 (purple in A).


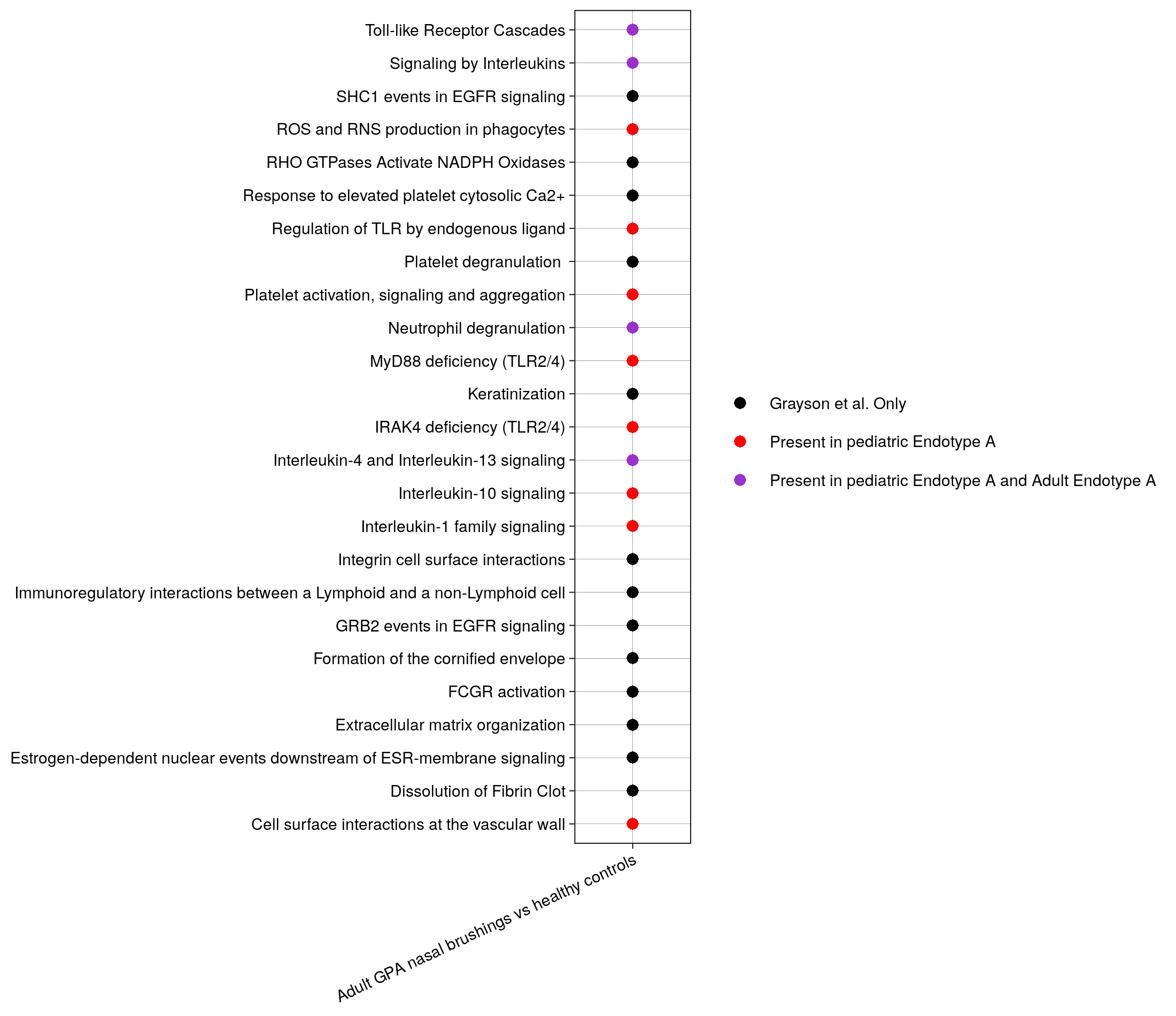


**C**


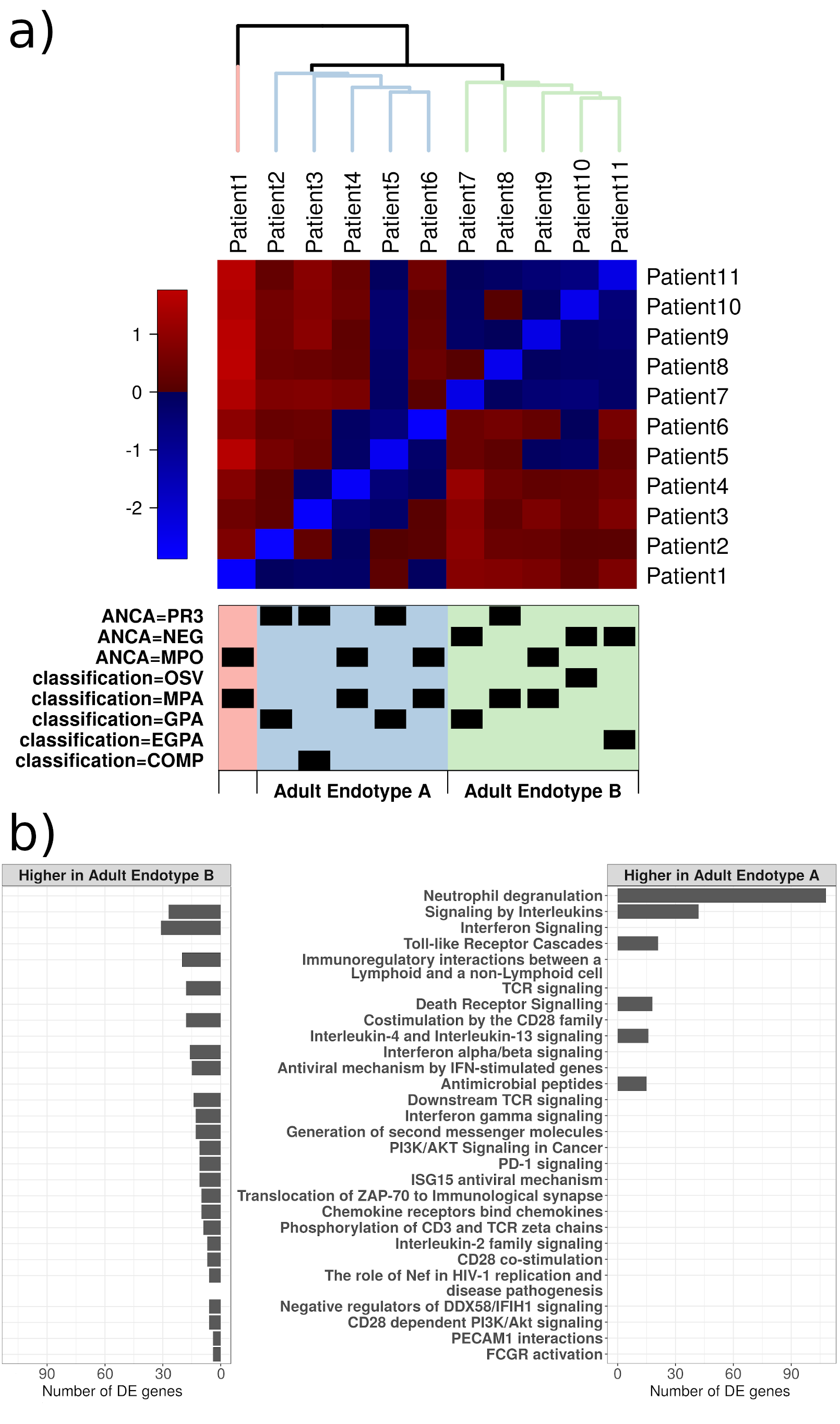


**A**


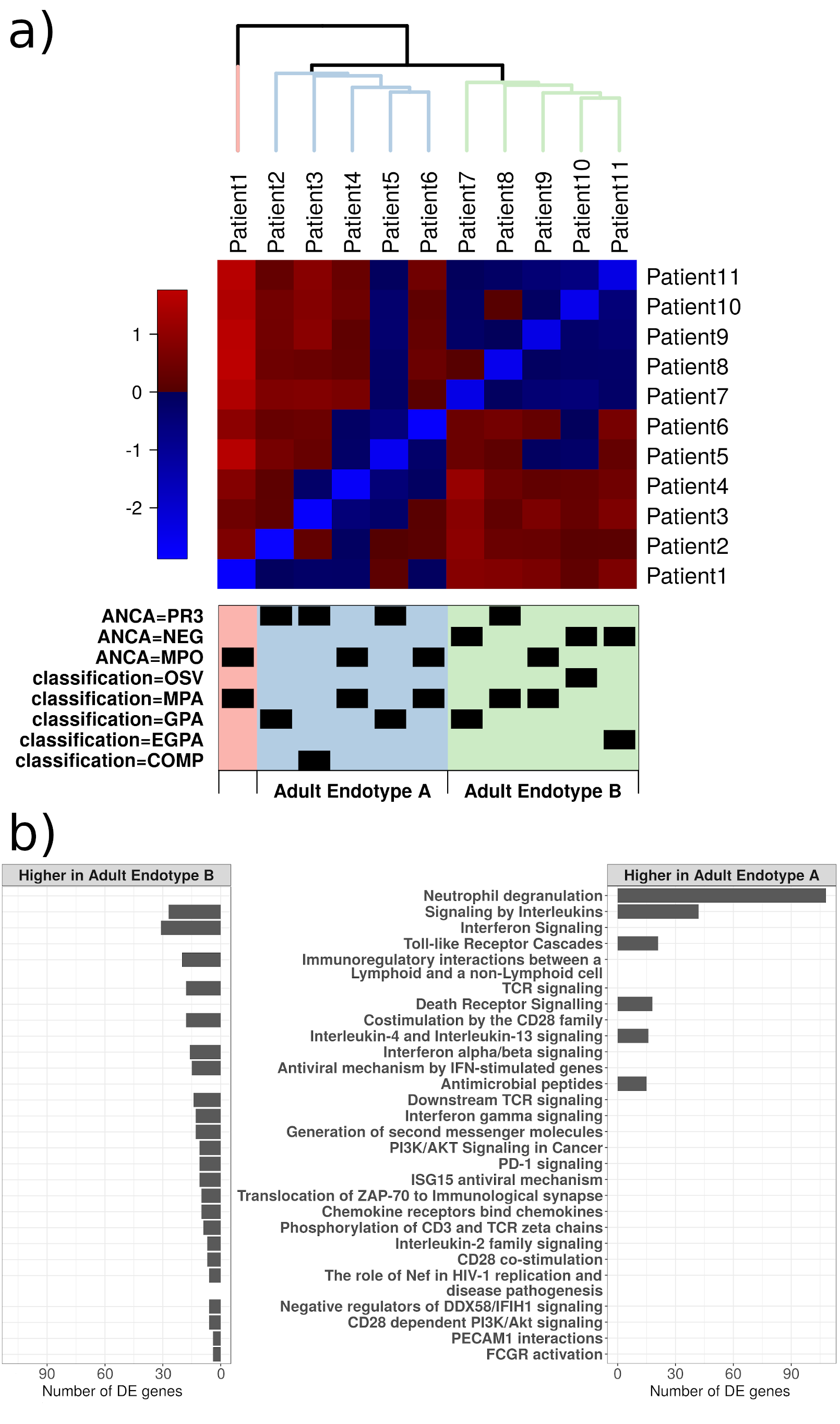


**B**

**Supplementary Figure 5:**  **Hierarchical clustering and dysregulated pathways in adults with small-to-medium sized vessel vasculitis. (A)** Hierarchical clustering (blue lines, Endotype A; green lines, Endotype B; red line, cluster 3) and heatmap of normalized gene expression (variance stabilized counts) based on RNA sequencing of whole blood in 11 adults with vasculitis (Table 2). (**B)** Reactome pathways enriched for differentially expressed genes (x-axis, total number of DE genes, FDR < 0.05, and fold change > 1.5) associated with adult patients in Endotype A or Endotype B. (**C)** Dot plot representing Reactome pathways enriched in differentially expressed genes identified by Grayson et al (*28*) between adult GPA nasal brushings and healthy control (black) and shared with pediatric Endotype A (red) or pediatric Endotype A and Adult Endotype A (purple).

**
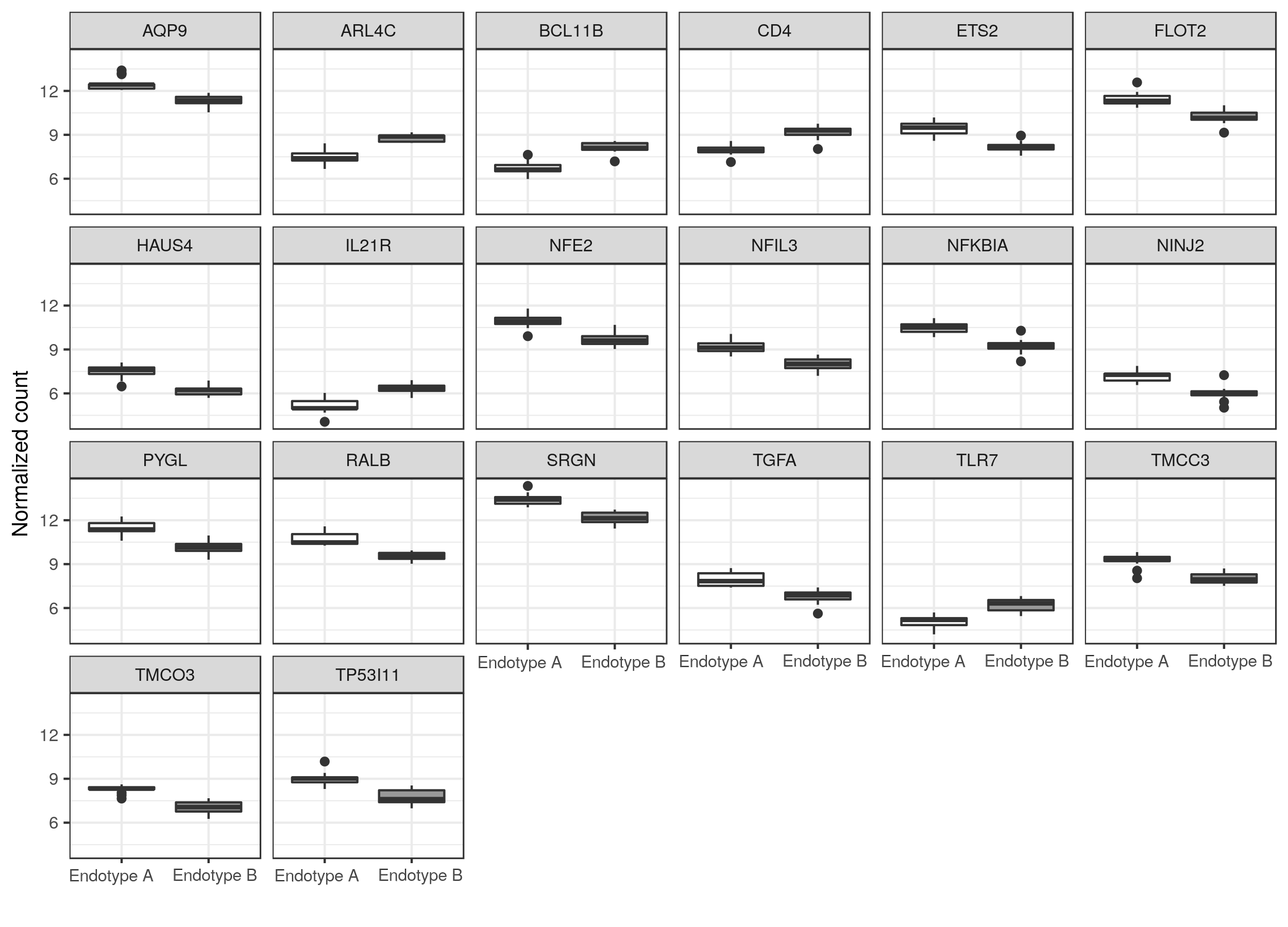
**

**Supplementary Figure 6: Normalized read counts of the 20 genes that discriminated samples into Endotype A and Endotype B.** Boxplots of normalized gene expression (variance stabilized counts) in samples belonging to each cluster.

**
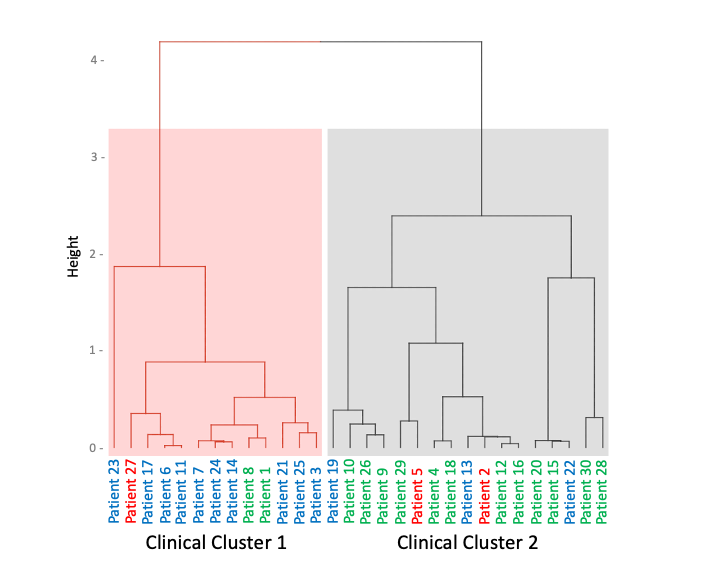
**

**Supplementary Figure 7: Unsupervised clustering of clinical characteristics for Cohort 1 pediatric patients.** Clinical metadata from children and adolescents with small-to-medium sized vessel vasculitis (n = 30, Cohort 1) was analyzed by hierarchical clustering using Multiple Factor Analysis (MFA) functions from FactoMineR [31]. Patients organized into two major clusters (x-axis), Clinical Cluster 1 and Clinical Cluster 2. Patient ID is colored as per endotype subclassification shown in Figure 1 where hierarchical cluster analysis was performed on RNA sequence data. Blue = Endotype A, green = Endotype B and red = Endotype Other. Clinical metadata used in the analysis excluding EMA classification is listed in Supplementary Table 2.

**Supplementary Table 1: Characteristics and classification of Cohort 2 pediatric vasculitis patients**

| **ID** | **^1^Cluster** | **^2^Endotype** | **^3^EMA** | **^4^ANCA** | **Time** | **Sex** |
| --- | --- | --- | --- | --- | --- | --- |
| C2 Patient 31 | one | Endotype A | GPA | PR3 | Relapse | Female |
| C2 Patient 32 | one | Endotype A | UCV | PR3 | Relapse | Female |
| C2 Patient 33 | two | Endotype B | GPA | MPO | Relapse | Female |
| C2 Patient 34 | two | Endotype B | MPA | BOTH | Relapse | Male |
| C2 Patient 35 | one | Endotype A | GPA | PR3 | Relapse | Female |
| C2 Patient 36 | two | Endotype B | GPA | MPO | Relapse | Female |
| C2 Patient 37 | one | Endotype A | uAAV | Not Done | Relapse | Female |
| C2 Patient 38 | other 1 | NA | PAN | NEG | Relapse | Male |
| C2 Patient 39 | two | Endotype B | MPA | MPO | Relapse | Male |
| C2 Patient 40 | other 2 | NA | uAAV | MPO | Relapse | Female |
| C2 Patient 41 | two | Endotype B | GPA | NEG | Relapse | Male |

**^1^**Hierarchical cluster based on RNA-sequence analysis ^2^Corresponding Endotype from Cohort 1 **^3^**European Medicines Agency classification. uAAV = unclassified AAV, UCV = unclassified (ANCA-negative) vasculitis. **^4^**PR3 and MPO indicate positivity for, respectively, anti-PR3 and anti-MPO antibodies. NEG means that neither anti-PR3 nor anti-MPO antibodies were detected and BOTH means that the individual was positive for PR3- and MPO-ANCA.

**Supplementary Table 2: Qualitative and quantitative metadata associated with hierarchical Endotype A and Endotype B**

| **Characteristic** | **Correlation (mean)^2^** | | **p-value** | |
| --- | --- | --- | --- | --- |
|  | **Endotype A** | **Endotype B** | **Endotype A** | **Endotype B** |
| EMA classification is MPA | none | positive | NS^1^ | 0.0365 |
| Received non-biologic, immune-suppressive drug treatment | none | positive | NS | 0.0365 |
|  |  | |  |  |
| Age at diagnosis (years) | positive (14) | none, (11) | 0.0145 | NS |
| Neutrophil count (x 10^9/L) | positive (10.4) | none (6.2) | 0.0181 | NS |
| PVAS total score | positive (21) | negative (14) | 0.0133 | 0.0061 |
| PVAS chest score | positive (4) | negative (1) | 0.0221 | 0.0022 |

^1^NS = not significant, p-value > 0.05.

^2^calculated mean within the cluster for numerical characteristics

Clinical data included sex, age at diagnosis, number of days to diagnosis, ANCA status (positivity for anti-MPO or anti-PR3 antibodies), EMA classification, absolute white blood cell (WBC) count, neutrophil count, eosinophil count, lymphocyte count, erythrocyte sedimentation rate (ESR), PVAS (and component ENT score, chest score and renal score), glomerular filtration rate (GFR), presence of hematuria, treatment, and serum concentrations of C-reactive protein (CRP) and hemoglobin (HGB). Treatment was categorized as oral corticosteroids, IV corticosteroids, plasma exchange, biologic immunosuppressive drugs, nonbiologic immunosuppressive drugs and other medications. Hematuria data were divided into one of three categories: < 5 red blood cells (RBC)/high power field (HPF), 5-10 RBC/HPF or >=10 RBC/HPF.

**Supplementary Table 3A: Reactome pathways enriched in the set of differentially expressed genes associated with Endotype A.** Differential expression between Endotypes A and B, FDR < 0.05, and fold change > 1.5. Enrichment measured with hypergeometric overlap, p-values adjusted with FDR.

| **Pathway Description** | **Genes in Dataset** | **Genes in Pathway** | **Adjusted p-value** |
| --- | --- | --- | --- |
| Neutrophil degranulation | 178 | 479 | 1.1x10^-56^ |
| Signaling by interleukins | 85 | 464 | 2.0x10^-05^ |
| Toll-Like Receptors Cascades | 38 | 157 | 9.8x10^-05^ |
| Toll-Like Receptor 4 (TLR4) Cascades | 33 | 131 | 0.00017 |
| Interleukin-4 and 13 signaling | 27 | 108 | 0.0012 |
| MyD88:Mal cascade initiated on plasma membrane | 25 | 96 | 0.0012 |
| Toll Like Receptor TLR6:TLR2 Cascade | 25 | 96 | 0.0012 |
| Cellular senescence | 40 | 195 | 0.0015 |
| Toll Like Receptor TLR1:TLR2 Cascade | 25 | 99 | 0.0015 |
| Toll-Like Receptor 2 (TLR2) Cascades | 25 | 99 | 0.0015 |
| Glycerophospholipid biosynthesis | 28 | 129 | 0.0085 |
| Oxidative Stress Induced Senescence | 27 | 125 | 0.011 |
| Signaling by NOTCH | 27 | 126 | 0.012 |
| TRAF6 mediated induction of NFkB and MAPK | 22 | 94 | 0.012 |
| Pre-NOTCH Expression and Processing | 14 | 47 | 0.012 |
| MyD88 deficiency (TLR2/4) | 6 | 10 | 0.012 |
| Toll Like Receptor 7/8 (TLR7/8) Cascade | 22 | 96 | 0.012 |
| MyD88 dependent cascade initiated on endosome | 22 | 96 | 0.012 |
| Phospholipid metabolism | 39 | 212 | 0.013 |
| Oncogene Induced Senescence | 11 | 33 | 0.015 |
| Toll Like Receptor 10 (TLR10) Cascade | 20 | 86 | 0.015 |
| Toll Like Receptor 5 (TLR5) Cascade | 20 | 86 | 0.015 |
| MyD88 cascade initiated on plasma membrane | 20 | 86 | 0.015 |
| Interleukin-6 signaling | 6 | 11 | 0.015 |
| IRAK4 deficiency (TLR2/4) | 6 | 11 | 0.015 |
| Diseases of immune system | 9 | 24 | 0.015 |
| Diseases associated with the TLR signaling | 9 | 24 | 0.015 |
| Growth hormone receptor signaling | 9 | 24 | 0.015 |
| Toll Like Receptor 9 (TLR9) Cascade | 22 | 100 | 0.015 |
| Toll Like Receptor 3 (TLR3) Cascade | 22 | 101 | 0.017 |
| MyD88-independent TLR4 cascade | 22 | 102 | 0.018 |
| TRIF(TICAM1)-mediated TLR4 signaling | 22 | 102 | 0.018 |
| Iron uptake and transport | 15 | 58 | 0.018 |
| Platelet activation, signaling, and aggregation | 44 | 262 | 0.018 |
| Cell surface interactions at the vascular wall | 27 | 137 | 0.018 |
| Cellular responses to stress | 65 | 428 | 0.018 |
| Rho GTPase cycle3 | 27 | 138 | 0.020 |
| Sema4D cell migration and growth-cone collapse | 8 | 21 | 0.021 |
| Signaling by Receptor Tyrosine Kinases | 67 | 448 | 0.021 |
| Interleukin-3, 5 and GM-CSF signaling | 13 | 48 | 0.022 |
| Fc-gamma receptor dependent phagocytosis | 19 | 86 | 0.025 |
| ROS, RNS production in phagocytes | 10 | 32 | 0.026 |
| Synthesis of PC | 9 | 28 | 0.037 |
| Interleukin-15 signaling | 6 | 14 | 0.043 |
| Signaling by Rho GTPases | 65 | 446 | 0.043 |
| NLR signaling pathways | 13 | 52 | 0.043 |
| TBC/RABGAPs | 12 | 46 | 0.043 |
| Interleukin-1 family signaling | 26 | 140 | 0.043 |
| Signal attenuation | 5 | 10 | 0.044 |
| Regulation of TLR by endogenous ligand | 7 | 19 | 0.045 |
| Death receptor signaling | 26 | 141 | 0.045 |

**Supplementary Table 3B: Reactome pathways enriched in the set of differentially expressed genes associated with Endotype B.**

| **Pathway Description** | **Genes in Dataset** | **Genes in Pathway** | **Adjusted p-value** |
| --- | --- | --- | --- |
| Translocation of ZAP-70 to immunological synapses | 14 | 19 | 1.2x10^-09^ |
| Generation of second messenger molecules | 18 | 33 | 1.2x10^-09^ |
| rRNA modification in the nucleus and cytosol | 23 | 62 | 1.7x10^-08^ |
| Phosphorylation of CD3 and TCR zeta chains | 13 | 22 | 1.9x10^-07^ |
| PD-1 signaling | 13 | 23 | 3.3x10^-07^ |
| Co-stimulation by the CD28 family | 22 | 70 | 8.6x10^-07^ |
| rRNA processing in the nucleus and cytosol | 40 | 196 | 9.4x10^-07^ |
| rRNA processing | 41 | 206 | 1.2x10^-06^ |
| rRNA processing in the nucleolus and cytosol | 37 | 186 | 5.6x10^-06^ |
| tRNA processing | 24 | 106 | 0.00011 |
| TCR signaling | 24 | 119 | 0.00077 |
| tRNA aminoacylation | 13 | 42 | 0.00077 |
| Downstream TCR signaling | 19 | 98 | 0.011 |
| Interferon signaling | 30 | 196 | 0.013 |
| tRNA processing in the nucleus | 13 | 56 | 0.017 |
| Immunoregulatory interactions between lymphoid and non-lymphoid cells | 22 | 132 | 0.026 |
| Nucleobase biosynthesis | 6 | 15 | 0.034 |
| CD28 co-stimulation | 9 | 33 | 0.037 |

**Supplementary Table 4A:** Reactome enrichment of genes of higher expression in relapse cluster 1. Differential expression between relapse clusters 1 and 2, FDR < 0.05, and fold change > 1.5. Enrichment measured with hypergeometric overlap, p-values adjusted with FDR.

| **Pathway Description** | **Genes in Dataset** | **Genes in Pathway** | **Adjusted p-value** |
| --- | --- | --- | --- |
| Neutrophil degranulation | 112 | 480 | 2.3x10^-34^ |
| Interferon signaling | 35 | 199 | 5.6x10^-06^ |
| Interferon alpha/beta signaling | 18 | 69 | 2.7x10^-05^ |
| Interferon gamma signaling | 21 | 92 | 2.7x10^-05^ |
| Toll-like Receptor Cascades | 28 | 155 | 3.7x10^-05^ |
| Growth hormone receptor signaling | 10 | 24 | 9.8x10^-05^ |
| Toll Like Receptor 4 (TLR4) Cascades | 24 | 129 | 9.9x10^-05^ |
| IRAK4 deficiency (TLR2/4) | 7 | 11 | 9.9x10^-05^ |
| Signaling by interleukins | 55 | 461 | 1.3x10^-04^ |
| MyD88:MAL(TIRAP) cascade initiated on plasma membrane | 19 | 95 | 3.3x10^-04^ |
| Toll Like Receptor TLR6:TLR2 Cascade | 19 | 95 | 3.3x10^-04^ |
| Toll Like Receptor TLR1:TLR2 Cascade | 19 | 98 | 3.9x10^-04^ |
| Toll Like Receptor 2 (TLR2) Cascade | 19 | 98 | 3.9x10^-04^ |
| Diseases of the Immune System | 9 | 24 | 3.9x10^-04^ |
| Diseases associated with the TLR signaling cascade | 9 | 24 | 3.9x10^-04^ |
| Rho GTPases Activate NADPH Oxidases | 9 | 24 | 3.9x10^-04^ |
| MyD88 deficiency (TLR2/4) | 6 | 10 | 4.9x10^-04^ |
| MyD88-independent TLR4 cascade | 18 | 99 | 0.0013 |
| TRIF(TICAM1)-mediated TLR4 signaling | 18 | 99 | 0.0013 |
| TRAF6 mediated induction of NFkB and MAP kinases upon TLR7/8 or 9 activation | 17 | 92 | 0.0017 |
| Toll Like Receptor 7/8 (TLR7/8) Cascade | 17 | 93 | 0.0018 |
| MyD88 dependent cascade initiated on endosome | 17 | 93 | 0.0018 |
| Toll Like Receptor 10 (TLR10) Cascade | 16 | 85 | 0.0018 |
| Toll Like Receptor 5 (TLR5) Cascade | 16 | 95 | 0.0018 |
| MyD88 cascade initiated on plasma membrane | 16 | 85 | 0.0018 |
| MAPK targets/Nuclear events mediated on MAP kinases | 9 | 31 | 0.0025 |
| Toll Like Receptor 9 (TLR9) Cascade | 17 | 97 | 0.0026 |
| Nuclear Events (kinase and transcription factor activation) | 8 | 25 | 0.0027 |
| Interleukin-4 and Interleukin-13 signaling | 18 | 108 | 0.0029 |
| Signaling by Receptor Tyrosine Kinases | 50 | 473 | 0.0030 |
| Regulation by IFNG signaling | 6 | 14 | 0.0030 |
| Interleukin-15 signaling | 6 | 14 | 0.0030 |
| Platelet activation, signaling and aggregation | 32 | 262 | 0.0039 |
| Toll Like Receptor 3 (TLR3) Cascade | 16 | 94 | 0.0047 |
| ERK/MAPK targets | 7 | 22 | 0.0064 |
| Interleukin-2 family signaling | 10 | 44 | 0.0066 |
| Interleukin-6 signaling | 5 | 11 | 0.0072 |
| Cellular Senescence | 25 | 195 | 0.0083 |
| Caspase activation via Death Receptors in the presence of ligand | 6 | 17 | 0.0083 |
| Death Receptor Signaling | 20 | 141 | 0.0083 |
| Signaling by NOTCH1 | 13 | 73 | 0.0097 |
| Regulation of TLR by endogenous ligand | 6 | 19 | 0.016 |
| Antigen processing-Cross presentation | 15 | 99 | 0.021 |
| Signaling by SCF-KIT | 9 | 43 | 0.021 |
| TP53 Regulates Transcription of Cell Death Genes | 9 | 44 | 0.024 |
| Interleukin-17 signaling | 12 | 72 | 0.026 |
| MAP kinase activation | 11 | 64 | 0.031 |
| Rho GTPase cycle | 18 | 138 | 0.036 |
| Spry regulation of FGF signaling | 5 | 16 | 0.038 |
| Role of LAT2/NTAL/LA8 on calcium mobilization | 5 | 16 | 0.038 |
| FOXO-mediated transcription of cell death genes | 5 | 16 | 0.038 |
| TRIF-mediated programmed cell death | 4 | 10 | 0.038 |
| Activation of the AP-1 family of transcription factors | 4 | 10 | 0.038 |
| Signal attenuation | 4 | 10 | 0.038 |
| Interleukin-3, Interleukin-15 and GM-CSF signaling | 9 | 48 | 0.038 |
| Activated NOTCH1 Transmits Signal to the Nucleus | 7 | 31 | 0.038 |

**Supplementary Table 4B:** Reactome enrichment of genes of higher expression in relapse cluster 2. Differential expression between relapse clusters 1 and 2, FDR < 0.05, and fold change > 1.5. Enrichment measured with hypergeometric overlap, p-values adjusted with FDR.

| **Pathway Description** | **Genes in Dataset** | **Genes in Pathway** | **Adjusted p-value** |
| --- | --- | --- | --- |
| L13a-mediated translational silencing of Ceruloplasmin expression | 21 | 111 | 1.1x10^-09^ |
| GTP hydrolysis and joining of the 60S ribosomal subunit | 21 | 112 | 1.1x10^-09^ |
| Eukaryotic Translation Initiation | 21 | 119 | 1.8x10^-09^ |
| Cap-dependent Translation Initiation | 21 | 119 | 1.8x10^-09^ |
| Formation of a pool of free 40S subunits | 19 | 101 | 4.5x10^-09^ |
| Eukaryotic Translation Elongation | 18 | 93 | 7.7x10^-09^ |
| rRNA processing | 26 | 205 | 7.8x10^-09^ |
| rRNA processing in the nucleus and cytosol | 25 | 195 | 1.3x10^-08^ |
| Major pathway of rRNA processing in the nucleolus and cytosol | 24 | 185 | 1.8x10^-08^ |
| Peptide chain elongation | 17 | 89 | 1.8x10^-08^ |
| Viral mRNA Translation | 17 | 89 | 1.8x10^-08^ |
| Translation | 30 | 291 | 2.7x10^-08^ |
| Selenoamino acid metabolism | 18 | 118 | 2.1x10^-07^ |
| Selenocysteine synthesis | 16 | 93 | 2.1x10^-07^ |
| Eukaryotic Translation Termination | 16 | 93 | 2.1x10^-07^ |
| Influenza Viral RNA Transcription and Replication | 19 | 134 | 2.1x10^-07^ |
| Nonsense Mediated Decay (NMD) independent of the Exon Junction Complex (EJC) | 16 | 95 | 2.8x10^-07^ |
| SRP-dependent co-translational protein targeting to membrane | 17 | 112 | 4.4x10^-07^ |
| Influenza Life Cycle | 19 | 144 | 6.1x10^-07^ |
| Influenza Infection | 19 | 155 | 1.9x10^-06^ |
| Nonsense-Mediated Decay (NMD) | 16 | 115 | 3.5x10^-06^ |
| Nonsense-Mediated Decay (NMD) enhanced by the Exon Junction Complex (EJC) | 16 | 115 | 3.5x10^-06^ |
| Generation of second messenger molecules | 9 | 33 | 6.3x10^-06^ |
| Translation initiation complex formation | 11 | 58 | 1.4x10^-05^ |
| Ribosomal scanning and start codon recognition | 11 | 58 | 1.4x10^-05^ |
| Activation of the mRNA upon binding of the cap-binding complex and eIFs, and subsequent binding to 43S | 11 | 59 | 1.6x10^-05^ |
| Formation of the ternary complex, and subsequently, the 43S complex | 10 | 51 | 3.1x10^-05^ |
| Signaling by ROBO receptors | 20 | 218 | 7.0x10^-05^ |
| Regulation of expression of SLITs and ROBOs | 17 | 171 | 1.3x10^-04^ |
| Translocation of ZAP-70 to the Immunological synapse | 6 | 19 | 2.3x10^-04^ |
| Metabolism of amino acids and derivatives | 26 | 372 | 3.2x10^-04^ |
| Infectious disease | 26 | 383 | 5.2x10^-04^ |
| TCR signaling | 12 | 119 | 0.0026 |
| Co-stimulation by the CD28 family | 9 | 70 | 0.0030 |
| Phosphorylation of CD3 and TCR zeta chains | 5 | 22 | 0.0066 |
| PD-1 signaling | 5 | 23 | 0.0080 |
| rRNA modification in the nucleus and cytosol | 7 | 62 | 0.034 |
| Branched-chain amino acid catabolism | 4 | 19 | 0.035 |
